# Antihypertensive Effects of Roselle-Olive Combination in L-NAME-Induced Hypertensive Rats

**DOI:** 10.1155/2017/9460653

**Published:** 2017-10-22

**Authors:** Rehab F. Abdel-Rahman, Alyaa F. Hessin, Marwan Abdelbaset, Hanan A. Ogaly, Reham M. Abd-Elsalam, Salah M. Hassan

**Affiliations:** ^1^Pharmacology Department, National Research Centre, Giza, Egypt; ^2^Chemistry Department, College of Science, King Khalid University, Abha, Saudi Arabia; ^3^Biochemistry Department, Faculty of Veterinary Medicine, Cairo University, Giza, Egypt; ^4^Pathology Department, Faculty of Veterinary Medicine, Cairo University, Giza, Egypt; ^5^Department of Biochemistry, Faculty of Science, Ain Shams University, Cairo, Egypt

## Abstract

This study aimed to evaluate the antihypertensive efficacy of a new combination therapy of *Hibiscus sabdariffa* and *Olea europaea* extracts (2 : 1; Roselle-Olive), using N(G)-nitro-L-arginine-methyl ester- (L-NAME-) induced hypertensive model. Rats received L-NAME (50 mg/kg/day, orally) for 4 weeks. Concurrent treatment with Roselle-Olive (500, 250, and 125 mg/kg/day for 4 weeks) resulted in a dose-dependent decrease in both systolic and diastolic blood pressure, reversed the L-NAME-induced suppression in serum nitric oxide (NO), and improved liver and kidney markers, lipid profile, and oxidative status. Furthermore, Roselle-Olive significantly lowered the elevated angiotensin-converting enzyme activity (ACE) and showed a marked genoprotective effect against oxidative DNA damage in hypertensive rats. Roselle-Olive ameliorated kidney and heart lesions and reduced aortic media thickness. Real-time PCR and immunohistochemistry showed an enhanced endothelial nitric oxide synthase (eNOS) gene and protein expression in both heart and kidney of Roselle-Olive-treated rats. To conclude, our data revealed that Roselle-Olive is an effective combination in which *H. sabdariffa* and *O. europaea* synergistically act to control hypertension. These effects are likely to be mediated by antioxidant and genoprotective actions, ACE inhibition, and eNOS upregulation by Roselle-Olive constituents. These findings provide evidences that Roselle-Olive combination affords efficient antihypertensive effect with a broad end-organ protective influence.

## 1. Introduction

Hypertension is a chronic medical condition in which the arterial blood pressure (BP) is elevated. It is a well-defined risk factor for many diseases such as coronary heart diseases, atherosclerosis, and stroke [1], in addition to kidney and cerebrovascular complications [2]. In the last decades, hypertension becomes one of the most common preventable causes of premature mortality worldwide. It is estimated to affect approximately 1 billion individuals, according to WHO [3], and contributes to about 12.8% of all annual deaths worldwide [4].

Modern medicine provides many classes of antihypertensive drugs. Nevertheless, target blood pressures are achieved in only a minority of patients in clinical practice. Limited efficacy of monotherapy to control hypertension especially in a patient with complications like diabetes mellitus, poor patient adherence to drug therapy, and occurrence of side effects are among the main reasons [5]. Therefore, people in the developing countries have been opted for herbal remedy as considerable alternatives to bridge the efficacy and therapeutic costs for the control of hypertension and its complications. Moreover, the lesser side effects and better tolerability direct the global attention towards the search for new drugs from natural sources [6].

Many of the naturally occurring medicinal plants have been reported for their hypotensive or antihypertensive effects [7]. However, more studies need to be done to elucidate the safety profile, to verify the effectiveness, and to explore the mechanisms of such herbal remedies. *Hibiscus subdariffa* L. (*HS*) is a bast fiber crop with edible calyx belonging to the family *Malvaceae* [8]. It is widely cultivated across tropical and subtropical areas for myriads of uses. Depending on geographical region, there are various vernacular names for *HS* as Karkadeh (Arabic), Roselle (English), Oseille de Guinée (French), and Bissap (Senegal) [8]. *HS* tea is used across the world as a hot drink or a nonalcoholic beverage and for a variety of health benefits [9]. Ethnomedicinal studies have valued roselle calyx extract for its pharmacological properties including hepatoprotective [10], nephroprotective [11], antioxidant [12], antihypertensive [13], antianaemic [14], antidiabetic [15], and diuretic [16]. *Olea europaea* L. (*OE*) or Olive, belonging to the family *Oleaceae*, is a small tree native to tropical and warm temperate regions of the world. Olive fruit is commercially important in the Mediterranean region as a prime source of olive oil [17]. In traditional medicine, olive leaves have been used for the treatment of atherosclerosis, hypertension, diabetes, wounds, and as diuretic [18]. The antihypertensive and cholesterol-lowering actions of olive leaves and its active substance, oleuropein, are well-documented [19, 20].

By the fact that hypertension possesses a multifactorial nature, combination therapy, using antihypertensive agents with various targets and/or mechanisms, may be theoretically favored. To date, relatively few studies have been done on natural antihypertensive remedy combinations. Although the antihypertensive effects of *HS* or *OE* have been reported individually [21, 22], their combination has not been studied yet. To the best of our knowledge, this study is the first to examine a combination of *H. sabdariffa* and *O. europaea* (Roselle-Olive) (2 : 1) against L-NAME-induced hypertension and to investigate whether “Roselle-Olive combination” would have synergistic antihypertensive potential to alleviate abnormalities in blood pressure, vascular dysfunction, oxidative stress, and end-organ damage in L-NAME-induced hypertensive rats and further to explore some aspects of its mechanism of action. Our hypothesis is that Roselle-Olive combination will integrate different mechanisms against hypertension. *H. sabdariffa* blocks the renin angiotensin pathway, and *O. europaea* fosters NO release and ROS inhibition; hence, augmented actions could be obtained. Besides, combining *HS* and *OE* therapies in Roselle-Olive could improve the cardiovascular outcomes with less dose of individual component, thus reducing any undesirable effects that may occur.

## 2. Materials and Methods

### 2.1. Chemicals and Reagents

L-NAME was purchased from Sigma Aldrich (Germany). RNA extraction kit and QuantiFast SYBR Green PCR kit were obtained from Qiagen (Hilden, Germany). Reverse transcription system was obtained from Thermo Scientific (Meridian Rd, Rockford, IL, USA). The chemicals 5,5-dithiobis-2-nitrobenzoic acid (DTNB), dihydrogen phosphate, trichloroacetic acid, thiobarbituric acid, and all other chemicals used in the experiment were of analytical grade and purchased from Sigma Chemical Company (St. Louis, MO, USA).

### 2.2. Preparation and Characterization of Roselle-Olive Combination

Roselle-Olive combination consists of 226 mg hydro alcoholic powdered extract from calyxes of *Hibiscus sabdariffa* and 113 mg hydro alcoholic powdered extract from leaves of *Olea europaea* as active ingredients. One milligram of Aerosil® 200 (inactive ingredient) was used as glidant. The extracts were obtained from Active Ingredients Company Sdn Bhd, Kuala Lumpur, Malaysia.

### 2.3. Animals

Adult male Sprague-Dawley rats (150–175 g) were used. All animals were housed under constant temperature and 12 h light/dark cycle. They were fed with standard chow diet. All animal procedures were performed in accordance with the recommendations of the National Institutes of Health (NIH) guide for care and use of laboratory animals (Publication number 85-23, revised 1985). Besides, the animals were treated according to the national guidelines stated by the ethical committee of the National Research Centre (NRC) and all experimental procedures were done according to the protocol approved by it. The experimental endpoint of this study was set when the scientific aims and objectives have been reached. During the experimental study, we ensured that pain and distress were minimized or relieved. At the end of the study, euthanasia or humane killing of rats was done by methods that induce rapid unconsciousness and death without pain or distress.

### 2.4. Single-Dose Toxicity of Roselle-Olive Combination

According to OECD guidelines (number 423), before conducting the toxicity experiment, the test substance, at doses that are recognized to result in marked pain or distress due to severely irritant properties, may need not to be administered. Moribund animals or animals clearly in pain or showing signs of enduring distress shall be humanely killed and are considered in the interpretation of the test results in the same way as animals that died on the test. Criteria for making the decision to kill moribund or severely suffering animals and guidance on the recognition of predictable or impending death are the subject of the guidance document on the recognition, assessment, and use of clinical signs as humane endpoints for experimental animals used in safety evaluation.

In this study, twelve adult male Sprague-Dawley rats (150–175 g), housed in polypropylene cages, were randomly allocated into two groups: the 1st group was kept as control and received DW orally and the 2nd group received an oral dose of freshly prepared 20% aqueous suspension of Roselle-Olive (5000 mg/kg). Treatment-related mortality or signs of toxicity were observed at day one at 0.5, 1, 2, 4, 8, 12, and 24 h from dosing then daily to day 14. The body weights of all animals were recorded daily. It can be concluded that administration of Roselle-Olive combination up to 5000 mg/kg could be considered safe. No mortality or morbidity was detected under the current experimental conditions. According to OECD guidelines, oral LD_50_ which is higher than 5000 mg/kg is considered to be safe. Roselle-Olive combination could be considered with a wide margin of safety for oral use. Since toxicity in humans cannot always be entirely extrapolated from animal studies, clinical evaluation should be performed to precisely define the safe dosage to advice in humans. For pharmacological studies, three doses were selected. One was a safe dose (1/10th of maximum tested safe dose = 500 mg/kg); the 2nd and the 3rd doses were lower than the safe dose (250 and 125 mg/kg).

### 2.5. Experimental Design

Hypertension was induced according to Majithiya et al. [23] by oral administration of L-NAME (0.5% *w*/*v*) in distilled water. Treatment was carried out as follows: group 1 (normal control) received only the vehicle (DW) orally; group 2 (hypertensive control) received L-NAME (50 mg/kg/day) orally for 4 successive weeks; group 3 (reference group) received L-NAME + lisinopril (10 mg/kg) for 4 successive weeks; and groups 4, 5, and 6 orally received L-NAME plus Roselle-Olive combination (500, 250, and 125 mg/kg/day), simultaneously for 4 successive weeks.

### 2.6. Blood Pressure Recording

BP (systolic and diastolic) and heart rate of conscious rats were measured at the start of the experiment and each week. Animals were restrained in the tubes for 10–20 min/day for 5 days prior to recording BP in the tail-cuff technique, and the animals were warmed for 30 min at 28°C in a thermostatically controlled heating cabinet (Ugo Basille, Italy) for better detection of tail artery pulse, where the tail was passed through a cuff and a tail-cuff sensor that was connected to an amplifier (ML 125 NIBP, AD Instruments, Australia). The amplified pulse was recorded during automatic inflation and deflation of the cuff. The mean arterial blood pressure (MAP) was calculated [24] using the following formula: MAP = DBP + 0.412(SBP − DBP).

### 2.7. Electrocardiography (ECG)

Rats were anesthetized by i.p. injection of 45 mg/kg thiopental [25]. ECG of rats was recorded for 1 min using ECG Powerlab module which consists of Powerlab/8sp and Animal Bio-Amplifier, in addition to Lab Chart 7 software for ECG analyzer.

### 2.8. Sampling

Twenty four hours after the last injection, animals were anesthetized with ethyl ether. Blood samples were collected by retro-orbital puncture and centrifuged at 4000 rpm/10 min to separate serum for measurement of biochemical indices. Afterward, all animals were sacrificed by cervical dislocation under ethyl ether anesthesia for humane reasons; the whole liver, kidney, and heart were immediately removed, rinsed in ice-cold normal saline, and kept at −80°C until further analyses. Parts of kidney and the heart tissues were placed in 10% neutral buffered formalin for histopathological examination.

#### 2.8.1. Biochemical Analyses

Serum samples were used to measure liver markers: alanine aminotransferase (ALT), aspartate aminotransferase (AST) according to the method of Reitman and Frankel [26], and gamma glutamyltransferase (GGT) according to Szasz, [27], and kidney markers: urea and creatinine according to the method of Wills and Savory [28] and Kroll et al. [29], respectively. Also, LPO expressed as MDA [30], NO (nitrate and nitrite, the end products of NO metabolism) [31], and reduced glutathione (GSH) [32] was estimated. ACE activity was measured using commercial ELISA kits. Lipid profile including total cholesterol, triglycerides, and high-density lipoprotein (HDL) was estimated by standard commercial kits (BioDiagnostics, Egypt). Low-density lipoprotein (LDL) was calculated by using Friedewald formula [33].

#### 2.8.2. Histopathological Examination

The kidneys, heart, and segment from thoracic aorta of the different groups were fixed and processed for obtaining 4 *μ*m paraffin embedding sections. The sections were stained with hematoxylin and eosin (H&E) and MT stain for assessment of fibrosis [34]. The histopathological evaluations of the glomeruli, tubules, and interstitial tissue of the kidney were performed according to Duarte et al. [35], using a scale of 0 to 4 as follows: 0 normal; 1 mild; 2 moderate; 3 severe; and 4 very severe.

The histopathological lesion scoring of the myocardium was performed according to Kanda et al. [36], using a scale from 0 to 4 as follows: 0 normal; 1 mild; 2 moderate; 3 severe; and 4 very severe. The percentage of the myocardial fibrosis (%) was performed as the mean of 10 fields/slide using Leica Qwin 500 Image Analyzer (Leica, Cambridge, England). The aorta tunica media thickness (from the internal to the external elastic lamellae) was measured in five sections of the thoracic aorta obtained from each group.

#### 2.8.3. Immunohistochemical Analysis

The immunohistochemical analysis of the kidney, heart, and aorta was done according to the methods described by Ogaly et al. [37]. The tissue sections were deparaffinized, rehydrated, and pretreated with 10 mM citrate buffer for antigenic retrieval. Sections were incubated for two hours at 4°C in a humidified chamber with one of the following primary antibodies: rabbit polyclonal anti-eNOS antibody diluted at 1 : 50 (Santa Cruz Biotechnology, USA) and the monoclonal anti-iNOS antibody diluted at 1 : 25 (Santa Cruz Biotechnology, USA). The tissue sections were incubated with a biotinylated goat anti rabbit and mouse antibody (Thermo Scientific, USA), streptavidin peroxidase (Thermo Scientific, USA), and 3,3′-diaminobenzidine tetrahydrochloride (DAB, Sigma). The slides were counterstained with Mayer's hematoxylin then dehydrated and mounted. Primary antibodies were replaced by PBS for negative controls. The stained sections were analyzed by Leica Qwin 500 Image Analyzer (Leica, Cambridge, England). In each field, the immunopositive area (dark brown) was recorded. Percentage of the positive stained area (%) was calculated as the mean of 10 fields/slide.

#### 2.8.4. Gene Expression Analysis by Quantitative Real-Time PCR

Gene expression analysis by quantitative real-time PCR was performed according to the method of Livak and Schmittgen [38]. In brief, total RNA was purified from 100 mg of tissue samples (the kidney and heart) using Qiagen RNeasy Mini kit following the manufacturer's protocol. Purity of the isolated RNA was detected spectrophotometrically (Thermo Scientific, USA). The purified RNA was reverse transcribed into cDNA and used for PCR with primers specific for eNOS and iNOS (Table 1). mRNA expression levels of the target genes were assessed using real-time PCR standardized by coamplification with GAPDH as a housekeeping gene, which served as an internal control. Real-time PCR was done in Biotechnology Unit, Faculty of Agriculture, Cairo University, Egypt. cDNA was added to a SYBR Green qPCR Master Mix (Qiagen) containing 30 pg/ml of each primer. The cDNA was amplified by 40 cycles of denaturation at 95°C for 15 s, annealing at 60°C for 15 s, and extension at 72°C for 45 s. During the first cycle, the 95°C step was extended to 1 min. The GAPDH gene was amplified in the same reaction to serve as the reference gene.

#### 2.8.5. DNA Damage Assessment by Comet Assay

Apoptotic changes in the heart and kidney were assessed using Comet assay. Comet assay was performed as described in [37]. Briefly, 100 mg of crushed kidney and heart samples was suspended in 1 ml ice-cold PBS, stirred for 5 min, and filtered. 100 *μ*l of cell suspension was thoroughly mixed with 600 *μ*l of low-melting agarose, followed by spreading of 100 *μ*l of the mixture on agarose precoated slides. The slides were left to solidify at 4°C, and then they were immersed in chilled lysing solution for 1 h at 4°C. The slides were removed and placed in a horizontal electrophoresis chamber, filled with freshly prepared electrophoretic alkaline buffer for 20 min. After electrophoresis, the slides were washed gently in 0.4 M Tris-HCl buffer and stained with ethidium bromide. The DNA migration patterns of 100 cells for each sample were observed using fluorescence microscope, and images were captured by a Nikon CCD camera. The qualitative and quantitative extent of DNA damage in the cells was estimated using the Comet 5 image analysis software developed by Kinetic Imaging Ltd. (Liverpool, UK).

### 2.9. Statistical Analysis

All results are expressed as means ± SE. Multiple group comparisons were performed by analysis of variance (ANOVA) followed by *LSD* test. Difference was considered significant when *p* < 0.05. GraphPad prism® software (version 6.00 for Windows, San Diego, California, USA) was used.

## 3. Results and Discussion

L-NAME-induced hypertension is a well-established experimental model characterized by generalized NO deficiency and progressive increase in BP if prolonged. As L-NAME model mimics hypertension in human, it is very suitable to study the cardiovascular effects of new agents [42]. The precise mechanism bases on the fact that L-NAME, a structural analog of L-arginine, is metabolize by nonenzymatic hydrolysis into the active form, N omega-nitro-L-arginine (L-NOARG), which competitively binds to endothelial NOS [43]. NOS inhibition attenuates both the synthesis and metabolism of NO, the smallest gaseous intercellular signaling molecule mediating the vascular relaxation. Subsequently, NO deficiency leads to systemic vasoconstriction and hypertension [44].

### 3.1. Toxicity Study

No mortality was recorded in the tested animals after 24 h of Roselle-Olive administration (5000 mg/kg). The study indicated that there were no significant alterations in animals' behavior or body weight after administering Roselle-Olive combination compared to normal control. Necropsy was done at day 14 after administration of Roselle-Olive, and no gross pathological changes were observed in comparison to healthy control animals.

### 3.2. Effect of Roselle-Olive Combination on Systolic BP (SBP), Diastolic BP (DBP), Pulse Rate, and the Mean Arterial Blood Pressure (MAP) in L-NAME-Induced Hypertensive Rats

**Figure 1:**
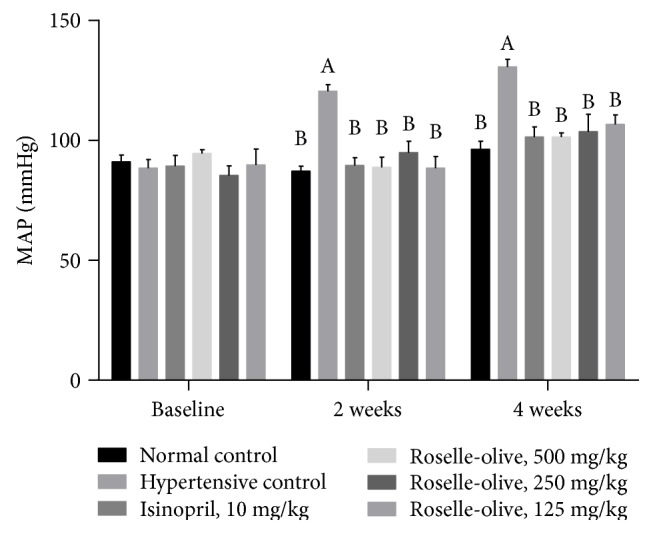
Effect of Roselle-Olive combination on MAP in L-NAME-induced hypertensive rats. Data were expressed as mean ± SE. of 5–7 experiments. Data were analyzed by one-way ANOVA followed by LSD for multiple comparison test, ^A^*p* < 0.05 versus normal control and ^B^*p* < 0.05 versus hypertensive rats.

In the current study, oral administration of L-NAME was associated with a significant rise in BP, MAP, and pulse rate compared with the normotensive control rats, validating the induction of hypertension. As presented in Figure 1, rats administered with L-NAME (50 mg/kg/day) showed higher SBP than normal control rats by 26% and 32%, after 15 and 30 days, respectively. Likewise, DBP was increased by 51% and 38.5%, after 15 and 30 days, respectively. Similarly, the heart rate of the L-NAME group was elevated by 50% and 40% after 15 and 30 days, respectively. These results are in agreement with previously described findings where L-NAME induced a prolonged increase in BP [45–47]. The observed high pulse rate in the hypertensive group could contribute to alterations in the vascular remodeling and progression of cerebral hypertrophy due to NO inhibition by L-NAME which in turn leads to derangement of sympathetic cardiovascular regulation [48, 49].

Administration of Roselle-Olive (500, 250, and 125 mg/kg) and lisinopril (a control hypotensive drug) caused a significant decline of BP and pulse rate in the hypertensive rats. Roselle-Olive (500, 250, and 125 mg/kg) decreased SBP in a dose-dependent manner by 17%, 15%, and 10%, after 30 days, respectively. Roselle-Olive (500, 250, and 125 mg/kg) decreased DBP by 26%, 25%, and 25%, after 30 days, respectively. This observed fall in BP in treated rats may be attributed to the hypotensive and vasorelaxant constituents of either *HS* such as anthocyanins, as delphinidin-3-O-sambubioside (hibiscin) and cyanidin-3-O-sambubioside (gossypicyanin) [50], or *OE* such as oleuropein, a major component of olive leaves [51]. Moreover, Aliyu et al. [52] suggested that *HS* hypotensive and heart rate dampening effects could be sympathetically mediated. Considering the previous studies that have claimed benefits of the individual components of our formula, *HS* and *OE,* on BP [22, 44, 50–53], the outcome of our study supports a synergistic antihypertensive and absence of competing mechanistic actions between the ingredients of the formula on arterial function activity of each individual ingredient.

### 3.3. Effect of Roselle-Olive Combination on ECG in L-NAME-Induced Hypertensive Rats

**Figure 2:**
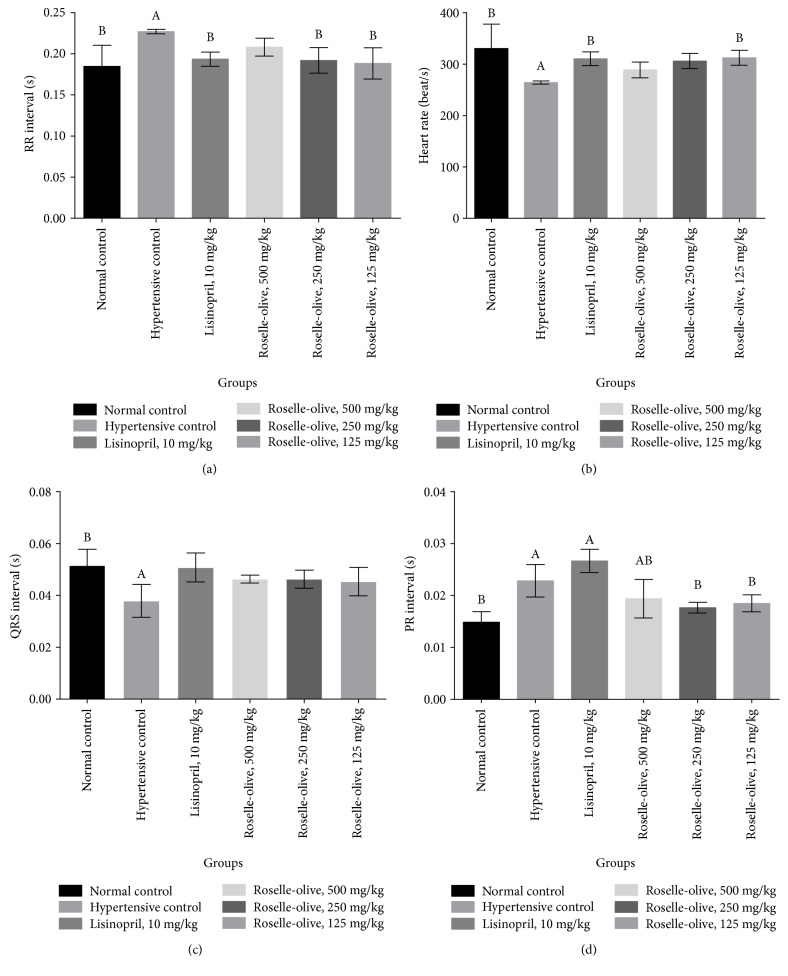
Effect of Roselle-Olive combination on ECG parameters (a) PR interval, (b) heart rate, (c) QRs interval, and (d) PR interval in L-NAME-induced hypertensive rats. Data were expressed as mean ± SE. of 5–7 experiments. Data were analyzed by one-way ANOVA followed by LSD for multiple comparison test, ^A^*p* < 0.05 versus normal control and ^B^*p* < 0.05 versus hypertensive rats.

**Figure 3:**
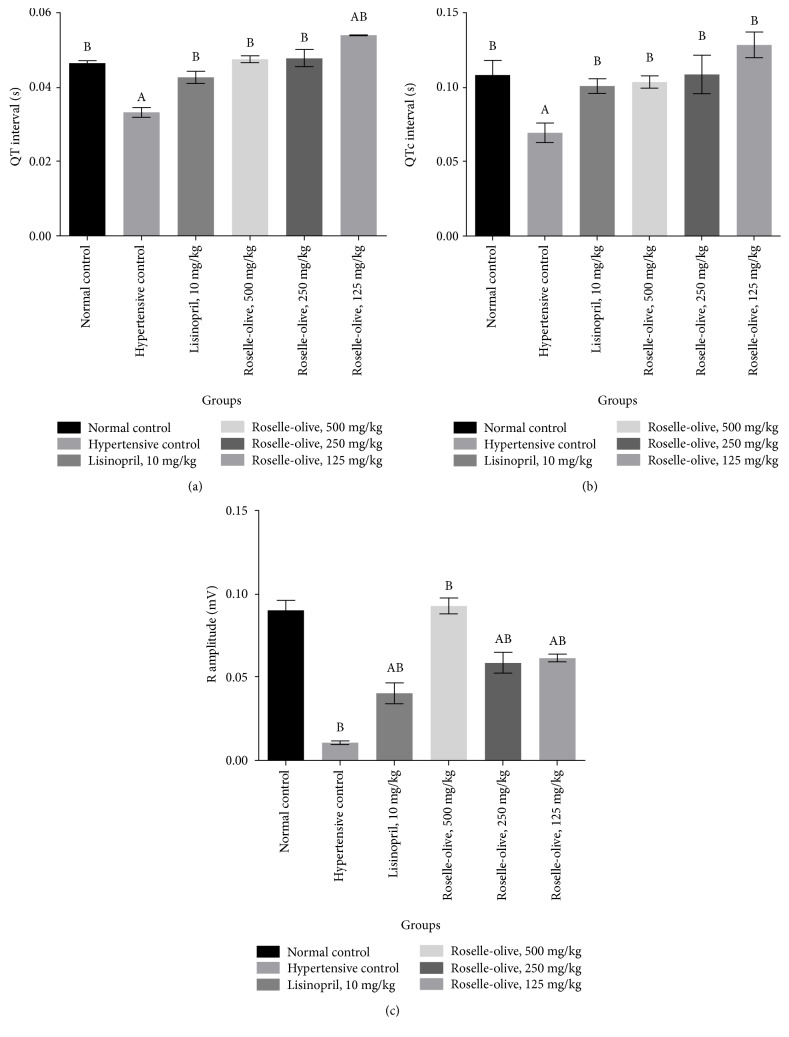
Effect of Roselle-Olive combination on ECG parameters (a) QT interval, (b) QTc interval, and (c) R amplitude in L-NAME-induced hypertensive rats. Data were expressed as mean ± SE. of 5–7 experiments. Data were analyzed by one-way ANOVA followed by LSD for multiple comparison test, ^A^*p* < 0.05 versus normal control and ^B^*p* < 0.05 versus hypertensive rats.

L-NAME increased the R-R and PR intervals. On the other hand, it decreased heart rate, QRS, QT, QT_c_, and R-amplitude as compared to normotensive rats. Impairment of ECG parameters by L-NAME goes in line with the findings of Chaswal et al. [54] and also could be attributed to the effect of the decreased NO production on vascular smooth tone [55]. Remarkably, lisinopril and all doses of Roselle-Olive ameliorated the L-NAME-induced changes in ECG parameters after 30 days of treatment (Figures 2 and 3).

### 3.4. Effect of Roselle-Olive Combination on Some Serum Biochemical Indices in L-NAME-Induced Hypertensive Rats

L-NAME-induced depletion of NO is associated with various end-organ damages mainly cardiac, renal, and vascular due to structural alterations in the microcirculation of these target organs [56, 57], in addition to oxidative stress which appears to play a prominent role in L-NAME-induced hypertension [58].

#### 3.4.1. Effect of Roselle-Olive Combination on Liver Markers in Hypertensive Rats

A strong body of evidences suggests that chronic inhibition of NOS by L-NAME alters various biochemical indices [59]. In the current study, the activities of the liver marker enzymes (ALT, AST, and *γ*-GT) were significantly elevated after L-NAME administration in comparison with normal controls, denoting hepatic injury (Table 2). These results are in line with previous studies [60]. Barón et al. [61] reported that, under physiological conditions, NO maintains hepatic perfusion and so inhibition of NO synthesis by L-NAME leads to a marked increase in perfusion pressure. Moreover, Cottart et al. [62] demonstrated that hepatic markers, including aminotransferases and hyaluronic acid, worsened with L-NAME-nonselective inhibition of NOS. Protective effects of Roselle-Olive against hypertension-associated liver injury were confirmed by the reduction in ALT and *γ*-GT serum levels. These data come in line with the previous data for the hepatoprotective effect of either *HS* [10, 63] or *OE* [22]. Interestingly, AST activity was not affected by Roselle-Olive treatment. This may be attributed to that AST is more diffusely represented extrahepatic in the heart, kidneys, brain, skeletal muscle, and red blood cells. Moreover, 80% of the cellular AST activity is localized in the mitochondria. Therefore, oxidative stress-associated mitochondrial injuries increase AST release [64].

#### 3.4.2. Effect of Roselle-Olive Combination on Kidney Markers in Hypertensive Rats

Creatinine and urea levels, the main kidney injury biomarkers, were significantly increased in the serum of L-NAME hypertensive rats. However, treatment with Roselle-Olive decreased both urea and creatinine levels (Table 2). Since NO acts as a key regulator of renal hemodynamics, the inhibited NO synthesis in hypertensive state leads to reduction in renal functions including impaired renal markers and decreased urinary sodium excretion along with reduced renal blood flow, urine flow rates, and glomerular filtration rates [65]. Former studies attributed the efficacy of *HS* as a potent diuretic agent to its vasorelaxant effect through elevation of NO production [18, 66]. In the same context, *OE* extract exhibited a protective effect against hypertension-associated renal impairment. The polyphenol constituents of olive leaf are known to have antihypertensive potential owning to their ability to enhance arterial dilatation via stimulation of endothelial NO production [67].

#### 3.4.3. Effect of Roselle-Olive Combination on Lipid Profile in Hypertensive Rats

Hypertension and hyperlipidemia are considered as two concomitant cardiovascular risk factors [68]. The results of the current study indicated dyslipidemia in L-NAME-hypertensive rats evidenced by elevated serum triglycerides and cholesterol and LDL coupled with decreased level of HDL compared to normal control group (Table 2). Concomitant administration of Roselle-Olive significantly modulated this dyslipidemic profile and nearly normalized the concentration of triglycerides, total cholesterol, LDL, and HDL (Table 2). However, the effect of *HS* on hypertensive-associated dyslipidemia is controversial. Several studies in animal models have stated the positive hypolipidemic effects of *HS* [69], while others have reported no significant effect on lipid profile parameters [70]. According to our finding, a combination of roselle and olive may contribute to the significant hypolipidemic effect observed in the treated groups. Together, the results of biochemical indices reflected the ability of the Roselle-Olive to reduce the incidence of liver and renal impairment and dyslipidemia associated with hypertension.

### 3.5. Effects of Roselle-Olive Combination on Angiotensin-Converting Enzyme (ACE) Activity in L-NAME-Induced Hypertensive Rats

Renin angiotensin system (RAS) components: renin, angiotensinogen, angiotensin-converting enzyme (ACE), and angiotensin II (Ang II) and its receptors, play an important role in the homeostatic control of arterial pressure, extracellular volume, and tissue perfusion [71]. ACE is a carboxypeptidase that catalyzes the conversion of Ang I into the bioactive Ang II [72]. NO antagonizes Ang II action on vascular tone and renal sodium excretion. While NO acts as vasodilator and natriuretic, Ang II acts as vasoconstrictor and active sodium-retaining hormone. Moreover, NO downregulates the synthesis of ACE. On the other hand, Ang II reduces NO bioavailability by promoting its oxidative degradation [73]. Functional imbalance between Ang II and NO is an important pathophysiologic mechanism involved in hypertensive end-organ damage [74]. Indeed, ACE inhibition is an important strategy in lowering BP and providing broad end-organ protection by attenuating oxidative stress and endothelial cell apoptosis [74]. In the present investigation, L-NAME stimulates ACE in the liver and kidney (Tables 3 and 4). These findings come in consistency with previous studies [73, 74]. Both Roselle-Olive and lisinopril exerted marked ACE inhibitory capacity in both hepatic and renal tissues of L-NAME hypertensive rats (Tables 3 and 4). These effects were involved in the mechanisms underlying *HS* antihypertensive influence [50, 75]. In the same context, the present results support the argument that *OE* reduced BP due to its vasorelaxant activity mediated by inactivation of ACE enzyme [76, 77]. Results of Roselle-Olive reflect the efficiency of this combination to lower blood pressure and concomitantly restore the homeostatic balance of vasoactive agents by inhibiting ACE which subsequently reduces Ang II and increases NO bioavailability [73]. Such a mechanism would be more effective in preventing or arresting end-organ disease [78].

### 3.6. Effects of Roselle-Olive Combination on Oxidative Stress Biomarkers and Antioxidant Profile in L-NAME-Induced Hypertensive Rats

Oxidative stress was found to be a primary cause in the pathogenesis of hypertension due to endothelial cell dysfunction [79]. Growing evidences suggest a crosslink between NO deficiency and development of oxidative stress in the onset and progression of vascular impairments [80]. During the normal physiology, NO reduces superoxide anion (O_2_^−^) production through a sustained suppression of NADPH oxidase, the major source of vascular oxygen radicals [81].

In line with [82, 83], our findings showed that administration of L-NAME is associated with increased production of reactive oxygen and nitrogen species (ROS/RNS) and subsequently oxidative stress in both liver and kidney. Malondialdehyde (MDA) is a prooxidant produced as secondary metabolite of lipid peroxidation (LPO) and indirectly reflects the oxidative degeneration of polyunsaturated fatty acids [84]. MDA was markedly accumulated in the hepatic and renal tissues of L-NAME group. Additionally, a marked depletion of endogenous antioxidants such as reduced glutathione (GSH) was observed (Tables 3 and 4). Our findings support the previous reports which suggest that the L-NAME BP-raising mechanism might not solely depend on NOS inhibition but may involve oxidative stress [45].

As presented in Tables 3 and 4, daily administration of Roselle-Olive was able to counteract the decrease in the antioxidant reserve in hypertensive rats. Roselle-Olive at the three dose levels (500, 250, and 125 mg/kg/day) restored GSH contents in renal and hepatic tissues and normalized MDA content in the liver, when compared to normal and lisinopril groups. Some studies considered the antioxidant and ROS scavenging abilities as another mechanism for hypotensive actions of *HS* [66, 84]. Additionally, olive was found to be a potent antioxidant and very effective in reducing ROS production [85, 86].

In addition to the inhibition of NO synthesis, L-NAME-induced oxidative stress results in inactivation of the available NO and inhibition of its vasodilator and natriuretic actions. Excessive O_2_^•−^ radicals interact with NO forming peroxynitrite (ONOO^−^) which exerts an additional detrimental effect on vascular function [74, 87]. This accelerated oxidative degradation of NO by ROS can worsen its inhibited production [88]. In the other hand, during chronic elevation in arterial blood pressure, NO formation from nitrate is stimulated and catalyzed by cytochrome P_450_ reductase. However, under oxidative stress, this NOS-independent NO production is switched off by ROS [89]. This view supports the concept that oxidative stress plays a key role in the endothelial dysfunction accompanying hypertension. Accordingly, the observed increase in NO bioavailability in Roselle-Olive-treated groups in face of its inhibited synthesis by L-NAME could be explained by the antioxidant activity and ROS scavenging action of Roselle-Olive combination together with its inhibitory action on ACE.

### 3.7. Effects of Roselle-Olive Combination on Histopathology of the Kidney and Heart in L-NAME-Induced Hypertensive Rats

#### 3.7.1. Histopathology of the Kidneys

**Figure 4:**
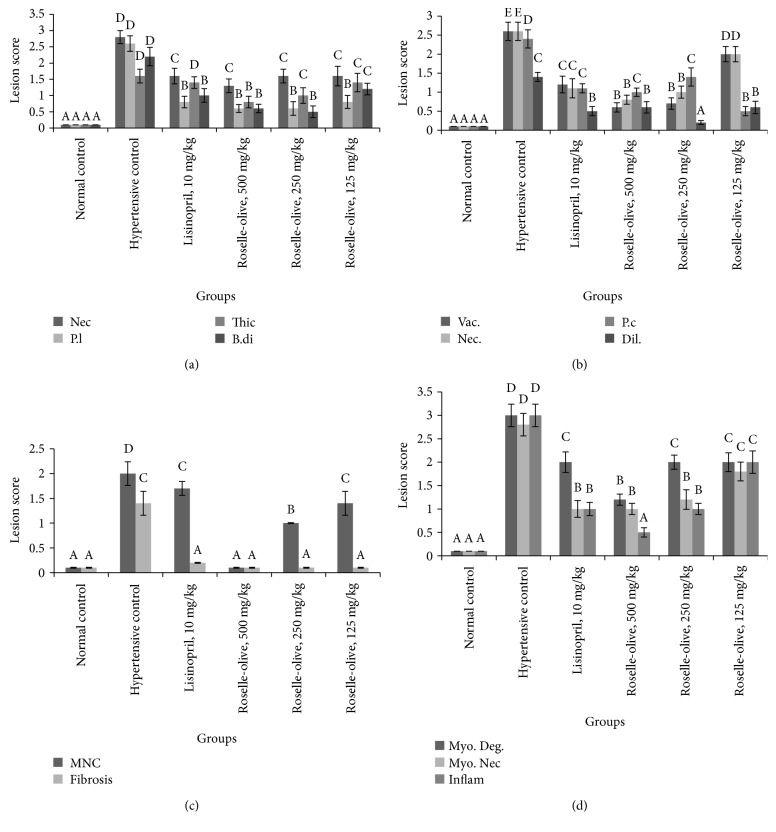
Effect of Roselle-Olive combination on kidney and myocardium lesion scoring in L-NAME-induced hypertensive rats. (a) Renal glomerular lesions; (b) renal tubular lesions; (c) renal interstitium lesions; (d) myocardium lesions. Values are presented as means ± SE. Mean values with different letters are significantly different (*p* < 0.05). Nec.: necrosis; P.l: protenious leakage; Thic: thickening of Bowman's capsule; B.di: Bowman's space dilation; Vac.: vacuolation; P.c: protein cast; Dil: tubular dilation; MNC: mononuclear cell; Myo. Deg.: myocardial degeneration; Myo. Nec.: myocardial necrosis; Inflam: inflammation.

**Figure 5:**
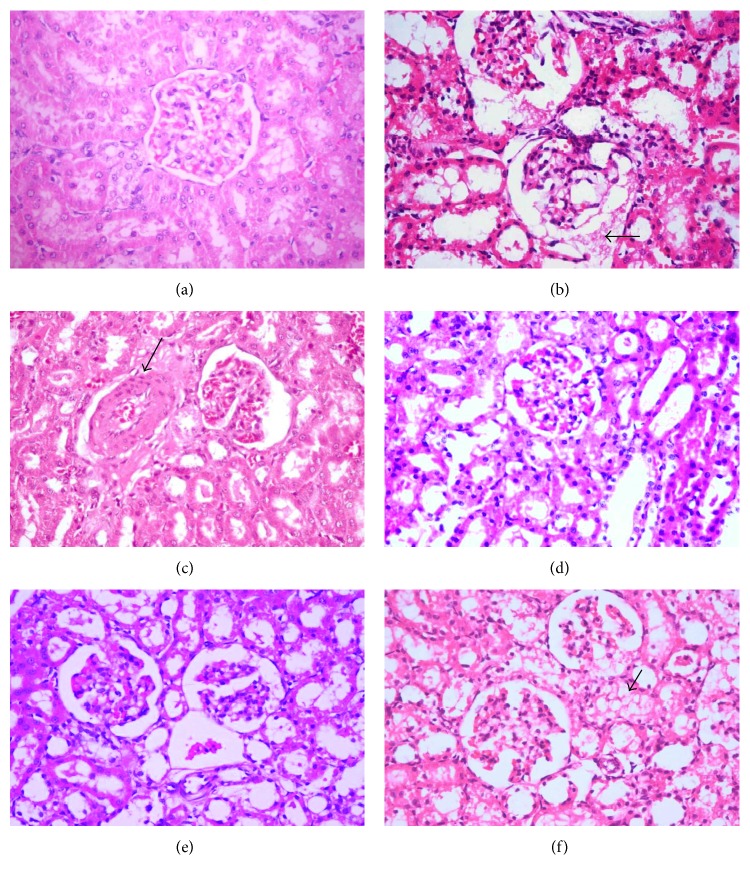
Histopathological changes of the kidneys in different experimental groups (H&E ×400). (a) Control normotensive group showing normal histological features. (b) L-NAME group showing marked necrosis of glomerular tuft, adhesion of glomerular tuft to parietal layer of the Bowman's capsule, the dilation of Bowman's space, and proteinous leakage into the space (arrow) with marked necrobiotic changes of renal tubules. (c) L-NAME + lisinopril group (10 mg/kg) showing mild glomerular necrosis, dilation of Bowman's space, partial glomerular tuft adhesion, slight thickening of the parietal layer of the Bowman's capsule, and moderate medial hypertrophy of blood vessels (arrow) with mild necrobiotic changes of renal tubular epithelium. (d) L-NAME + Roselle-Olive (500 mg/kg) group showing moderate necrobiotic changes of renal tubules (arrow). (e) L-NAME + Roselle-Olive (250 mg/kg) group showing mild glomerular tuft necrosis, adhesion of glomerular tuft, dilation of Bowman's space, and proteinous leakage. (f) L-NAME + Roselle-Olive (125 mg/kg) group showing moderate glomerular changes marked necrobiotic changes of renal tubules (arrow).

The histopathological evaluation of the kidneys in rats of different groups was summarized in Figures 4(a), 4(b), and 4(c). The kidneys of the normotensive group revealed no histopathological changes (Figure 5(a)). The kidneys of L-NAME hypertensive group showed significant increased lesion scoring compared to the normal control group (Figures 4(a), 4(b), and 4(c)). These lesions included glomerular hypertrophy with mesangiolysis that is characterized by necrosis of mesangial and endothelial cells, adhesion of glomerular tuft to a parietal layer of the Bowman's capsule, dilation of Bowman's space with proteinous leakage, and moderate thickening of the parietal layer of the Bowman's capsule. Moreover, the renal tubular epithelia showed marked necrobiotic changes with extensive proteinous cast accumulation in their lumina. Collagen depositions with mononuclear inflammatory cell aggregation in the interstitial tissue were also observed. L-NAME-induced renal histopathological changes were markedly attenuated in all Roselle-Olive and lisinoprinil-treated groups (Figures 5(c), 5(d), 5(e), and 5(f)). Thereby, treatment with Roselle-Olive at different doses significantly improved the renal histopathological findings.

Emerging evidence supports a unique role of local RAS in the kidney response to hypertensive stimuli and the induction of hypertension [90]. Increased Ang II mediates the arterial and glomerular hypertension and increases nephron glomerular filtration rate and nephron blood flow leading to nephron damage and subsequent renal failure [90]. Ang II exerts its effects by increasing ROS generation and release of proinflammatory chemokines causing various glomerular injuries [91]. On the basis of kidney biochemical and pathological findings (Tables 2 and 4, Figures 4 and 5), it seems fair to suggest that the ameliorative effect of Roselle-Olive on L-NAME-induced renal damage and reduction of renal fibrosis is mediated through its antioxidant action and inhibition of RAS through inhibition of ACE. These results provide confirmatory evidence that ACE inhibitors are able to prevent the intraglomerular vascular action that resulted from renal hypertension and glomerular hyperfiltration more effectively than other antihypertensives [92].

#### 3.7.2. Histopathology of the Heart

**Figure 6:**
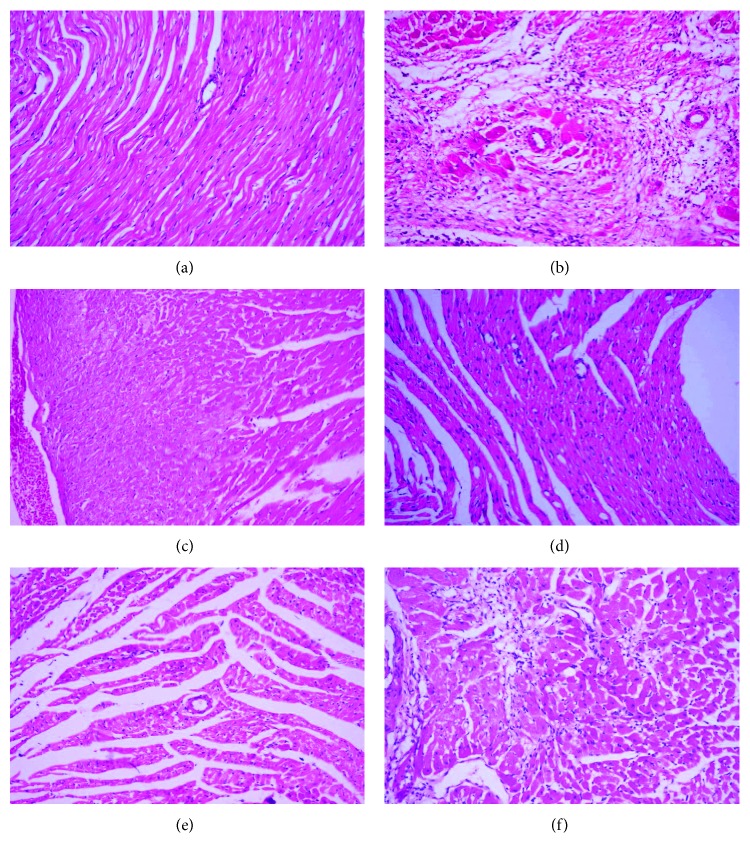
Photomicrograph of heart stained with H&E stain (×200). (a) Control normotensive group showing normal histological features. (b) L-NAME group showing extensive myocardial fibrosis, myocardial loss, and mononuclear inflammatory cell infiltration. (c) L-NAME + lisinopril group (10 mg/kg) showing moderate myocardial degeneration. (d) L-NAME + Roselle-Olive (500 mg/kg) group and (e) L-NAME + Roselle-Olive (250 mg/kg) group showing mild myocardial degeneration. (f) L-NAME + Roselle-Olive (125 mg/kg) group showing moderate myocardial degeneration, necrosis, mononuclear inflammatory cell aggregation, and collagen fiber deposition.

**Figure 7:**
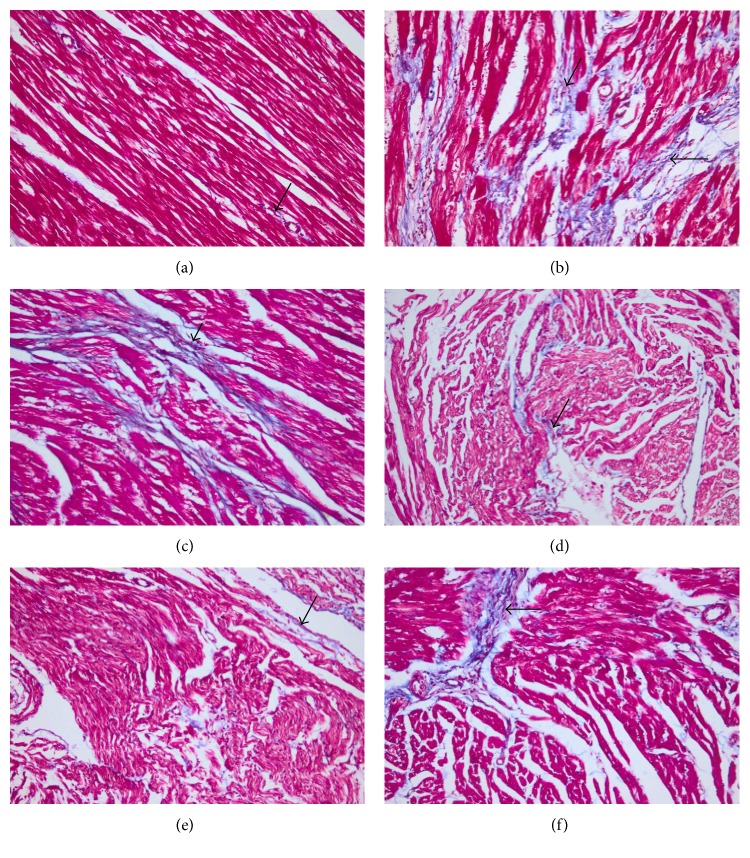
Photomicrograph of the heart stained with MT stain (×200). (a) Control normotensive group showing normal collagen fiber deposition around coronary blood vessels (arrow). (b) L-NAME group showing extensive collagen fiber deposition (arrow). (c) L-NAME + lisinopril group (10 mg/kg) showing moderate collagen fiber deposition (arrow). (d) L-NAME + Roselle-Olive (500 mg/kg) group and (e) L-NAME + Roselle-Olive (250 mg/kg) group showing slight collagen fiber deposition (arrow) in the interstitial tissue. (f) L-NAME + Roselle-Olive (125 mg/kg) group showing moderate myocardial fibrosis (arrow).

**Figure 8:**
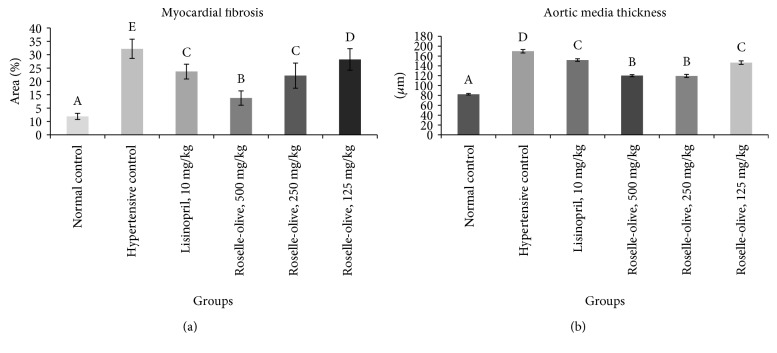
Effect of different treatment on the heart. (a) Myocardial fibrosis; (b) aortic tunica media thickening. Values are presented as means ± SE. Mean values with different letters are significantly different (*p* < 0.05).

The histopathological lesion scoring of the heart of the different groups was summarized in Figure 4(d). The heart of the normotensive group showed a normal histological finding of the myocardium (Figure 6(a)). The L-NAME hypertensive group revealed severe myocardial degeneration, necrosis, and fibrosis with mononuclear cell infiltration (Figure 6(b)). Roselle-Olive and lisinoprinil-treated groups showed a significant reduction in all myocardial lesions as shown in Figures 4(d) and 6. In Masson's trichrome- (MT-) stained sections, the heart of L-NAME hypertensive rats showed extensive collagen fiber deposition (Figure 7(b)) and increased myocardial fibrosis % (Figure 8(a)) compared to the normal controls. Groups that received Roselle-Olive at the three dose levels (500, 250, and 125 mg/kg/day) showed marked attenuation of myocardial fibrosis (Figures 7(c), 7(d), 7(e), 7(f), and 8(a)). Previous studies demonstrated that NO-deficient hypertension by L-NAME resulted in marked cardiac inflammation and development of myocardial fibrosis due to a significant increase in the cardiac density of macrophage and T-cell that produce several cytokines that promote fibroblast proliferation and collagen deposition [93, 94]. Roselle-Olive combination, similar to ACE inhibitor treatment, reduced the macrophage infiltration and cellular proliferation [95] and reduced myocardial fibrosis.

The results of the aortic media thickness were summarized in Figure 8(b). The aorta of the control group showed normal histological feature of tunica intima, tunica media, and tunica adventitia. The L-NAME hypertensive group showed focal tunica intima thickening and a significant increase in the tunica media thickness comparing to the control group. Roselle-Olive administration resulted in a significant reduction in the tunica media thickness when compared with the hypertensive group (Figure 8(b)).

### 3.8. Effects of Roselle-Olive Combination on Nitric Oxide Level and Nitric Oxide Synthase (eNOS and iNOS) Protein and Gene Expression in L-NAME-Induced Hypertensive Rats

NO enzymatic synthesis from L-arginine is derived from three different synthases: endothelial (eNOS), inducible (iNOS), and neuronal (nNOS) synthases [96]. For maintenance of BP homeostasis, NO, as endogenous vasodilator, translocates into vascular smooth muscle cells (VSMCs) and initiates a series of chemical interactions beginning with activation of soluble guanylate cyclase which then catalyzes the cyclization of guanosine triphosphate (GTP) to its cyclic form (cGMP). Increased cGMP level then causes a decrease in intracellular Ca2^+^ and subsequent vasodilatation [97]. Moreover, NO action on VSMCs inhibits the adhesion of leukocytes and platelets to the endothelial lining and impeding the proinflammatory state. Therefore, alteration in NOS function or expression is a candidate in the pathogenesis of hypertension.

**Figure 9:**
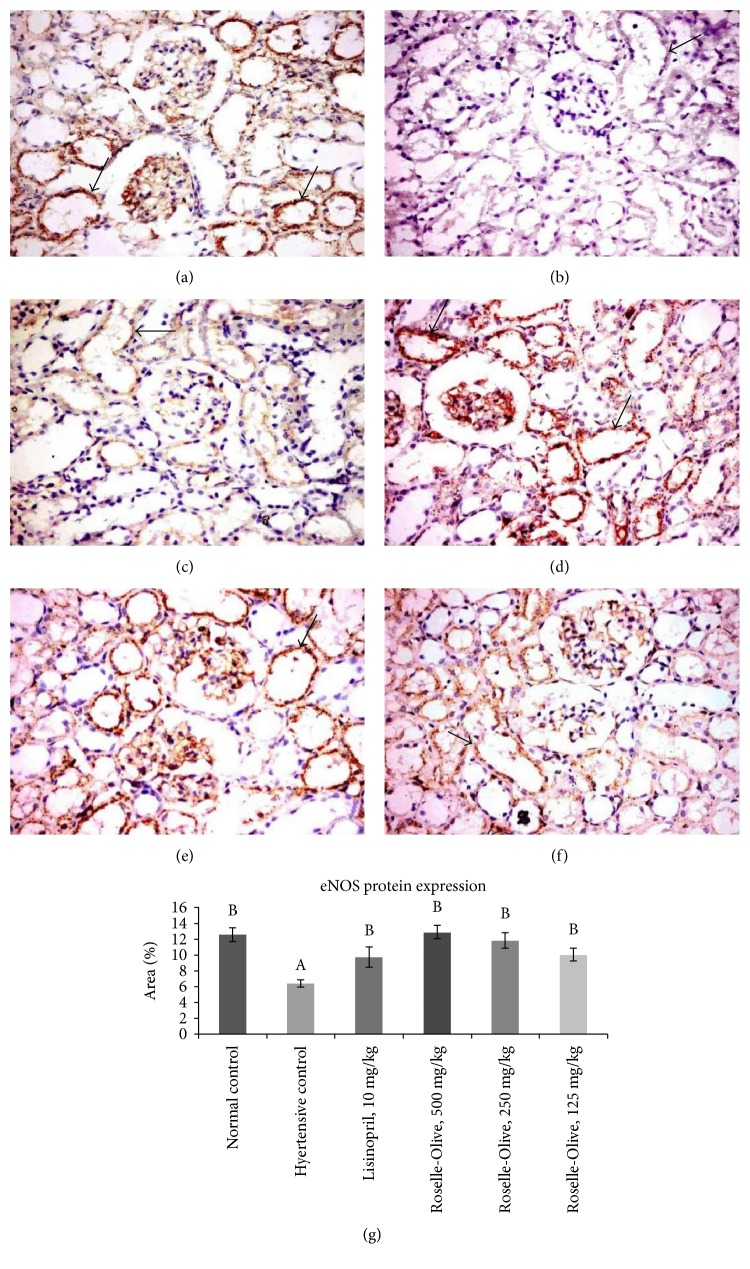
eNOS immunohistochemistry in the kidney tissues of different experimental groups (×400). The eNOS immunoreactivity was characteristically cytoplasmic and the cytoplasm was stained brown color (arrow), (a) control normotensive group moderate immunoreactivity. (b) L-NAME group showing very weak immunopositive reaction. (c) L-NAME + lisinopril group (10 mg/kg) showing moderate immunoreactivity. (d) L-NAME + Roselle-Olive (500 mg/kg) group showing strong immunoreactivity. (e) L-NAME + Roselle-Olive (250 mg/kg) group showing strong immunoreactivity. (f) L-NAME + Roselle-Olive (125 mg/kg) group showing moderate immunoreactivity. (g) Bar chart represents the eNOS immunopositivity expressed as area %. Mean values with different superscripts are significantly different (*p* < 0.05).

**Figure 10:**
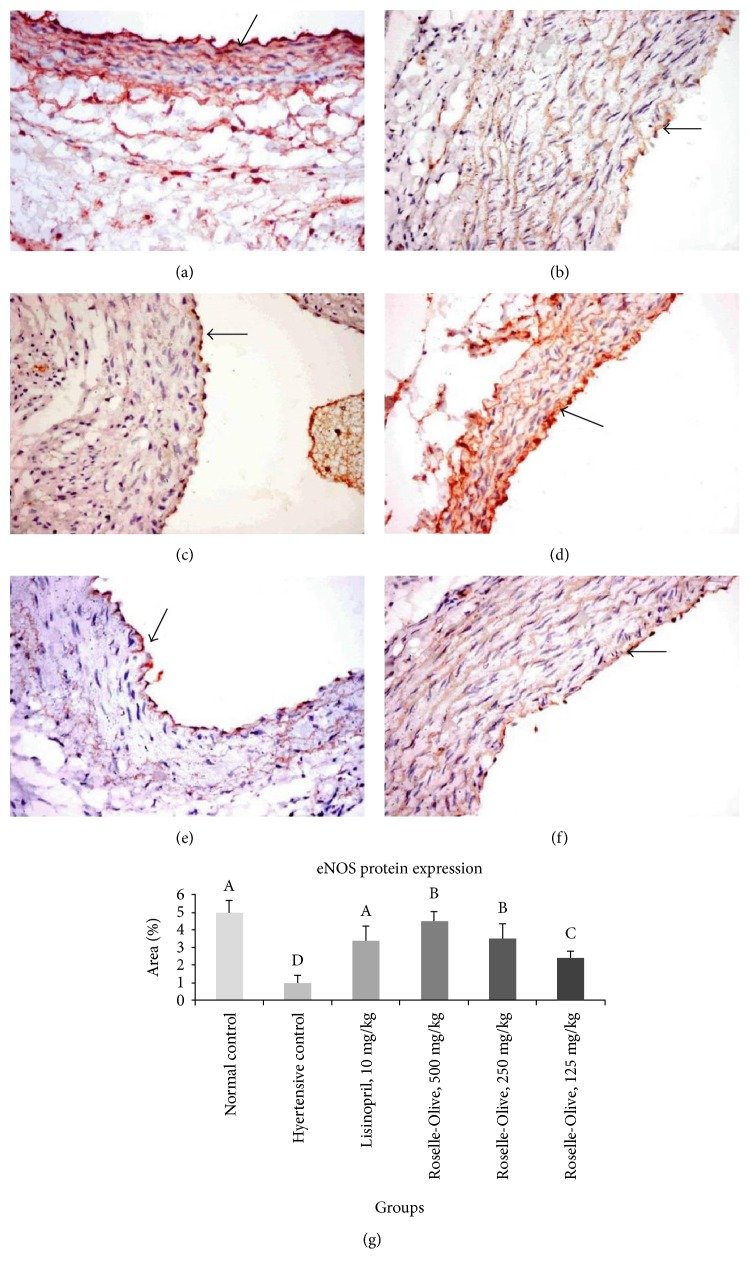
eNOS immunohistochemistry in the aorta of different experimental groups (×400). The eNOS immunoreactivity was in the endothelial lining blood vessels (arrow), (a) control normotensive group strong immunoreactivity. (b) L-NAME group showing very weak immunostaining. (c) L-NAME + lisinopril group (10 mg/kg) showing moderate immunoreactivity. (d) L-NAME + Roselle-Olive (500 mg/kg) group showing strong immunopositive reaction. (e) L-NAME + Roselle-Olive (250 mg/kg) and (f) L-NAME + Roselle-Olive (125 mg/kg) group showing moderate immunoreactivity. (g) Bar chart represents the eNOS immunopositivity expressed as area %. Mean values with different superscripts are significantly different (*p* < 0.05).

eNOS is the constitutively expressed isoform in the vascular endothelium and serves as the predominant source of NO for the regulation of vascular tone. Thereby, eNOS has been proven to have numerous protective effects in the cardiovascular system, while eNOS impairment or knockout leads to hypertension [98]. Despite much less known about nNOS in human, nNOS-generated NO may play an important role in the physiologic regulation of the basal vasomotor tone and blood flow [99]. During inflammation, a substantial production of NO has been found to be derived from iNOS that was induced by the inflammation [37]. In the present study, NO production was inhibited in the liver and kidney of the L-NAME hypertensive group to about 63% and 42% of its normal levels, respectively. NO levels were markedly restored in both liver and kidney of the lisinopril-treated groups (Tables 3 and 4). All dose levels of Roselle-Olive were efficient to nearly normalize NO level in the liver of hypertensive rats, while in the kidney, a dose-dependent increase in NO levels to 51%, 52%, and 65% of its normal levels in Roselle-Olive-treated groups (125, 250, and 500 mg/kg), respectively (Tables 3 and 4). Our results on both mRNA and protein levels indicated a downregulation of eNOS in the L-NAME hypertensive group and their positive responses to Roselle-Olive or lisinoprinil treatment. At the gene level, eNOS mRNA levels in the kidney and heart of the hypertensive group were reduced to about one tenth, 0.14-fold and 0.16-fold, respectively (Table 5). The treatment of rats with lisinopril resulted in restoration of eNOS expression. Administration of Roselle-Olive showed a protective effect against L-NAME-induced eNOS downregulation to be increased to 3.9-fold and 0.7-fold in the kidney and 2.9-fold and 10-fold in the heart of the Roselle-Olive-treated groups (500 and 250 mg/kg), respectively (Table 5). At protein level, immunohistochemistry revealed that eNOS was expressed mainly in renal tubular epithelial cells, endothelial lining capillary tuft, and endothelial lining of aorta, renal, and cardiac blood vessels with a significant decrease in eNOS immune-staining in the kidney (Figure 9), heart, and aorta (Figure 10) of the L-NAME group compared to the normotensive group. eNOS protein expression showed a significant increase in lisinopril and Roselle-Olive-treated groups compared to the hypertensive group (Figures 9 and 10). Restoration of eNOS expression confirmed by the increased hepatic and renal NO contents (Tables 3 and 4) and the subsequent vasodilatation and reversing the pathologic arterial remodeling. These results suggest upregulation of eNOS as a mechanism mediating the antihypertensive action of Roselle-Olive combination. The present data supports previous findings demonstrating that olive pomace improves endothelial dysfunction in hypertensive animals by enhancing eNOS expression [100]. Moreover, *HS* was found to ameliorate hypertension by mechanisms associated with endothelium-derived NO-cGMP pathway and inhibition of Ca2^+^ influx into VSMCs [101, 102].

**Figure 11:**
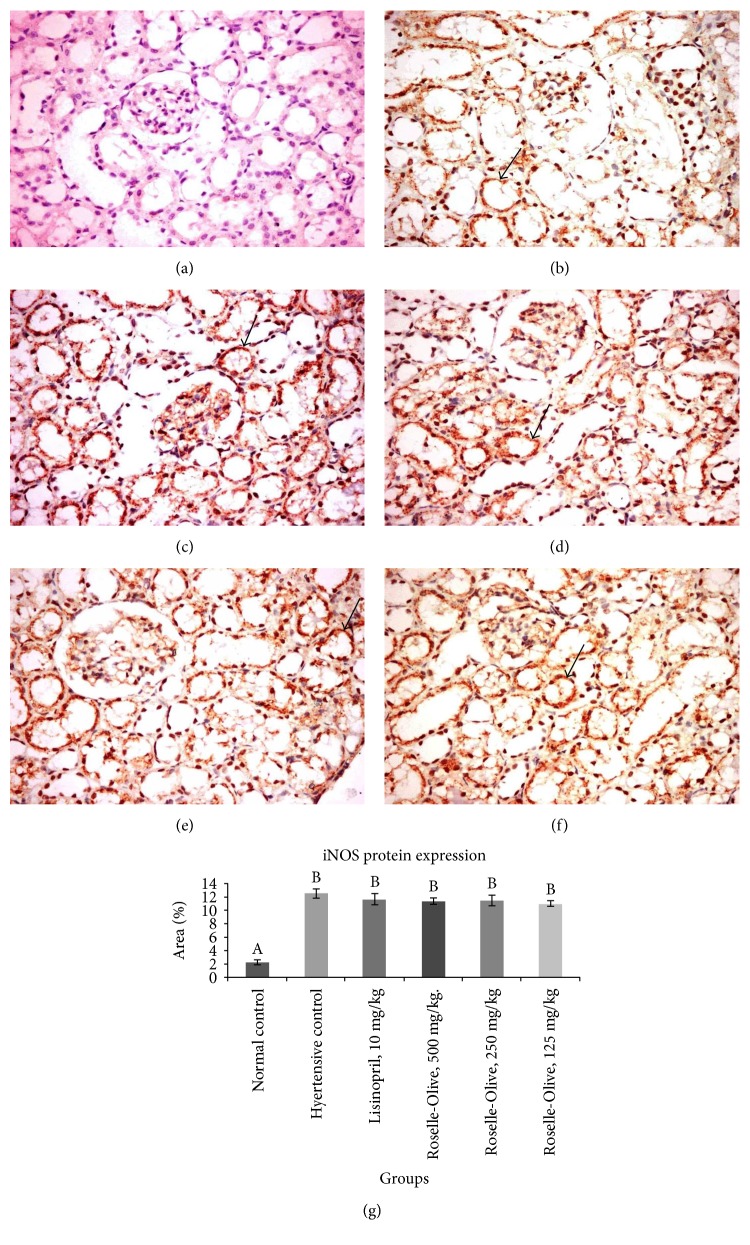
iNOS immunohistochemistry in the kidney tissues of different experimental groups (×400). (a) Control normotensive group showing very weak immunostaining. (b) L-NAME group showing strong immunostaining. (c) L-NAME + lisinopril group (10 mg/kg), (d) L-NAME + Roselle-Olive (500 mg/kg) group, (e) L-NAME + Roselle-Olive (250 mg/kg) group, and (f) L-NAME + Roselle-Olive (125 mg/kg) group showing strong immunostaining. (g) Bar chart represents the iNOS immunopositivity expressed as area %. Mean values with different superscripts are significantly different (*p* < 0.05).

Regarding iNOS expression at mRNA and protein levels, we observed that iNOS mRNA did not show significant differences between the examined groups (Table 6), whereas on the protein level, all L-NAME-treated groups displayed enhanced iNOS expression in the kidneys and heart compared to control group (Figure 11). These findings agreed with Pechánová et al. [103] that in L-NAME hypertension, increased iNOS was observed to serve as a major source of hemodynamically essential NO production. The discrepancy between iNOS mRNA and protein levels in our study may be explained by the fact that proteins undergo regulations apart from gene transcription [104]. Considerable evidences demonstrate that increased iNOS expression contributes to the pathogenesis of hypertension. Excessive NO derived by iNOS upregulation reacts with superoxide radicals forming peroxynitrite which promotes nitrosative stress and endothelial impairment. Additionally, excessive iNOS activity upregulates arginase activity that competes with eNOS for L-arginine for NO formation. This accompanied with eNOS uncoupling with enhanced superoxide anion production instead of NO [105].

### 3.9. Effects of Roselle-Olive Combination on DNA Damage in L-NAME-Induced Hypertensive Rats

A strong association correlates between the excessive ROS/RNS generation during the pathogenesis of hypertension and the oxidative damage of most cell macromolecules including protein, lipids, and DNA [79]. Oxidative DNA damage is progressed by several mechanisms including the direct attack of ROS to DNA causing DNA base oxidation and deamination, binding of LPO end products to DNA, and damage of repair enzymes [106].

**Figure 12:**
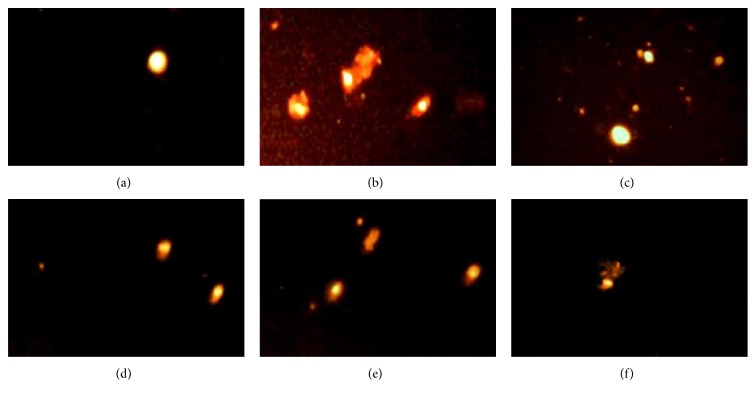
DNA damage in the kidney detected by comet assay in the different experimental groups. (a) Normal control group, (b) hypertensive (L-NAME) group, (c) L-NAME + lisinopril-treated group (10 mg/kg), (d) L-NAME + Roselle-Olive-treated group (500 mg/kg), (e) L-NAME + Roselle-Olive-treated group (250 mg/kg), and (f) L-NAME + Roselle-Olive-treated group (125 mg/kg).

**Figure 13:**
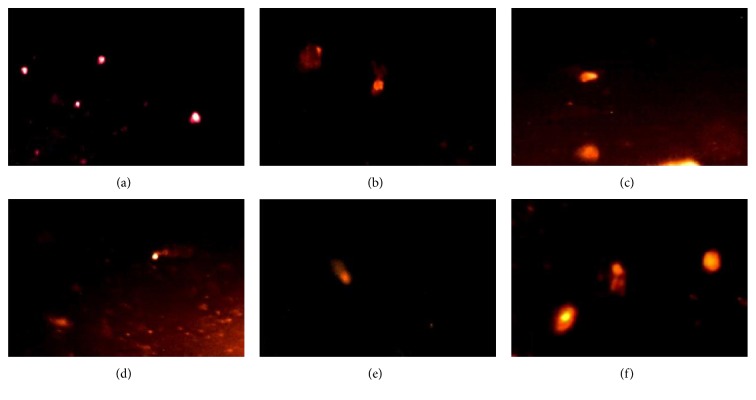
DNA damage in the heart detected by comet assay in the different experimental groups. (a) Normal control group, (b) hypertensive (L-NAME) group, (c) L-NAME + lisinopril-treated group (10 mg/kg), (d) L-NAME + Roselle-Olive-treated group (500 mg/kg), (e) L-NAME + Roselle-Olive-treated group (250 mg/kg), and (f) L-NAME + Roselle-Olive-treated group (125 mg/kg).

In the current study, comet assay was used as a well-validated technique to assess DNA fragmentation and damage [37]; L-NAME-induced hypertension was associated with a marked DNA damage in the kidney (Table 7, Figure 12) and heart (Table 8, Figure 13). The evidence for DNA damage is the significant elevation in the comet parameters, presented as tail length (*μ*m), tail DNA (%), and tail moment which was considered as the main indicative parameter used for DNA damage (Tables 7 and 8). Moreover, a comet-like tail implies the presence of a damaged DNA strand. The length of the tail increases with the extent of DNA damage as observed in the L-NAME hypertensive group (Figures 12 and 13). Moreover, a small comet head and a large broom-like tail were observed in the L-NAME hypertensive group. On the other hand, coadministration of lisinopril deteriorated the effect of L-NAME through significant reduction of the tail length, damaged DNA %, tail moment, and reducing the intensity of comet tail (Tables 7 and 8). Administration of Roselle-Olive showed a dose-dependent protective effect against L-NAME-induced oxidative DNA damage in the kidney and heart as shown in Tables 7 and 8, respectively.

The probable mechanism underlying the genoprotective potential of Roselle-Olive as observed in our study could be by virtue of its powerful antioxidant capacity and its ability to mitigate excess ROS and LPO. These data were in line with previous reports on effect either *HS* or *OE* against DNA damage. Ghosh et al. [107] showed that the antigenotoxic property of *HS* extract is presumably attributed to its antioxidant properties. Additionally, previous *in vitro* studies showed that *HS* is genoprotectant against oxidative DNA damage to cultured hematopoietic stem cells [108]. Besides, many studies demonstrated a potent genoprotective effect of olive leaf extract or phenols against DNA oxidative damage [103, 104]. Although, conflicting results were obtained in other studies in which olive showed no significant effect on DNA damage [109].

To the best of our knowledge, this study is the first to examine a combination of *H. sabdariffa* and *O. europaea* (Roselle-Olive) against L-NAME-induced hypertension at three dose levels. Roselle-Olive combination tried to integrate two different mechanisms against hypertension. *H. sabdariffa* blocks the renin angiotensin pathway, and *O. europaea* fosters NO release and ROS inhibition; hence, augmented actions could be obtained. Roselle-Olive inhibited the ACE activity by 85%, respectively, when compared to the L-NAME group. Moreover, Roselle-Olive combination restored eNOS expression and subsequently maintained the vasoprotective NO level. The significant antioxidant potential with the genoprotective effect against apoptotic DNA was found to participate in mediating Roselle-Olive antihypertensive and end-organ protective actions. Together, these outcomes placed Roselle-Olive combination as a promising therapeutic agent against hypertension and its associated complication.

## 4. Conclusion

The present study provides evidences that the new formula “Roselle-Olive” exhibited a considerable antihypertensive potential at the three examined dose levels (500, 250, and 125 mg/kg). Roselle-Olive combination significantly normalized the elevated systolic and diastolic BP as well as the pulse rate after two and four weeks of treatment. Roselle-Olive was efficient to improve liver and kidney functions and reversed the dyslipidemic effect of L-NAME-induced hypertension as well. Moreover, the present study suggested a multimechanistic action mediating the Roselle-Olive antihypertensive effect including antioxidant, genoprotective, and ACE inhibitory action in addition to upregultation of eNOS expression. These findings point out the augmented beneficial effects of Roselle-Olive combination as a complementary treatment in the management of hypertension and its complications.

## Figures and Tables

**Table 1:** Primer sequences for the studied genes.

Gene		Primer sequence	Reference
eNOS	F	TACGGAGCAGCAAATCCAC	[39]
	R	CAGGCTGCAGTCCTTTGATC	

iNOS	F	GGAGCGAGTTGTGGATTG	[40]
	R	CCAGGAAGTAGGTGAGGG	

GADPH	F	ACCACAGTCCATGCCATCAC	[41]
	R	TCCACCACCCTGTTG CTGTA	

**Table 2:** Effect of Roselle-Olive combination on some serum biochemical parameters in L-NAME-induced hypertensive rats.

Groups	ALT (U/l)	AST (U/l)	*γ*-GT (U/l)	Urea (mg/dl)	Creatinine (g/dl)	Triglycerides (mg/dl)	Cholesterol (mg/dl)	HDL (mg/dl)	LDL (mg/dl)
Group 1	35.1 ± 2.86^ǂ^	67.3 ± 3.08^ǂ^	6.2 ± 0.39^ǂ^	37.6 ± 2.60^ǂ^	0.5 ± 0.04^ǂ^	78.6 ± 3.09^ǂ^	67.9 ± 3.14^ǂ^	47.6 ± 3.16^ǂ^	10.5 ± 0.77^ǂ^
Group 2	95.4 ± 4.35^∗^	117.9 ± 4.32^∗^	13.4 ± 1.06^∗^	63.9 ± 3.58^∗^	0.9 ± 0.09^∗^	105.0 ± 4.47^∗^	81.7 ± 3.35^∗^	27.3 ± 1.61^∗^	25.7 ± 1.97^∗^
Group 3	50.5 ± 3.38^∗^^ǂ^	83.3 ± 3.09^ǂ^	12.4 ± 0.61^∗^	78.2 ± 3.09^∗^^ǂ^	0.9 ± 0.06^∗^	94.9 ± 3.08^∗^	73.9 ± 3.24	23.1 ± 1.85^∗^	22.4 ± 1.60^∗^
Group 4	60.4 ± 1.55^∗^^ǂ^	113.3 ± 5.84^∗^	7.9 ± 0.64^ǂ^	35.6 ± 2.42^ǂ^	1.0 ± 0.09^∗^	75.0 ± 3.77^ǂ^	64.4 ± 3.47^ǂ^	42.6 ± 2.41^ǂ^	8.3 ± 0.90^ǂ^
Group 5	52.3 ± 3.54^∗^^ǂ^	100.9 ± 5.60^∗^	8.5 ± 0.61^ǂ^	34.8 ± 2.69^ǂ^	0.9 ± 0.08^∗^	83.1 ± 3.61^ǂ^	64.6 ± 3.80^ǂ^	55.4 ± 1.00^ǂ^	9.7 ± 0.62^ǂ^
Group 6	53.4 ± 2.59^∗^^ǂ^	107.1 ± 4.50^∗^	5.0 ± 0.52^ǂ^	33.2 ± 0.79^ǂ^	0.9 ± 0.14^∗^	82.7 ± 2.62^ǂ^	63.9 ± 3.12^ǂ^	54.1 ± 1.45^ǂ^	8.5 ± 0.36^ǂ^

Group 1: normal control; group 2: hypertensive control (L-NAME 50 mg/kg); group 3: L-NAME + lisinopril (10 mg/kg); group 4: L-NAME + Roselle-Olive (500 mg/kg); group 5: L-NAME + Roselle-Olive (250 mg/kg); group 6: L-NAME + Roselle-Olive (125 mg/kg). Values are expressed as mean ± SE (*n* = 7). ^∗^*P* < 0.05, statistically significant from the normal control group at the corresponding time. ^ǂ^*P* < 0.05, statistically significant from the hypertensive control group at the corresponding time.

**Table 3:** Effect of Roselle-Olive combination on ACE activity, NO, and oxidative status in the liver of L-NAME-induced hypertensive rats.

Groups	Liver homogenate			
	ACE activity (ng/g)	NOx (*μ*mol/l)	GSH (*μ*mol/g)	MDA (nmol/g)
Group 1	1.9 ± 0.23^ǂ^	74.8 ± 6.5^ǂ^	7.4 ± 0.62^ǂ^	85.7 ± 5.48^ǂ^
Group 2	8.8 ± 0.29^∗^	47.6 ± 1.62^∗^	4.7 ± 0.23^∗^	126.7 ± 9.38^∗^
Group 3	1.3 ± 0.12^ǂ^	83.7 ± 3.26^ǂ^	6.4 ± 0.43	80.3 ± 4.01^ǂ^
Group 4	1.3 ± 0.06^ǂ^	67.7 ± 6.09^ǂ^	6.6 ± 0.21^ǂ^	82.8 ± 1.96^ǂ^
Group 5	1.4 ± 0.04^ǂ^	65.1 ± 4.94^ǂ^	6.0 ± 0.22	86.2 ± 1.88^ǂ^
Group 6	1.9 ± 0.05^ǂ^	70.8 ± 2.72^ǂ^	6.0 ± 0.64	82.2 ± 5.22^ǂ^

Group 1: normal control; group 2: hypertensive control (L-NAME 50 mg/kg); group 3: L-NAME + lisinopril (10 mg/kg); group 4: L-NAME + Roselle-Olive (500 mg/kg); group 5: L-NAME + Roselle-Olive (250 mg/kg); group 6: L-NAME + Roselle-Olive (125 mg/kg). Values are expressed as mean ± SE (*n* = 7). ^∗^*P* < 0.05, statistically significant from the normal control group at the corresponding time. ^ǂ^*P* < 0.05, statistically significant from the hypertensive control group at the corresponding time.

**Table 4:** Effect of Roselle-Olive combination on ACE activity, NO, and oxidative status in the kidney of L-NAME-induced hypertensive rats.

Groups	Kidney homogenate			
	ACE activity (ng/g)	NOx (*μ*mol/l)	GSH (*μ*mol/g)	MDA (nmol/g)
Group 1	2.0 ± 0.14^ǂ^	121.1 ± 6.29^ǂ^	8.1 ± 0.31^ǂ^	78.4 ± 1.65^ǂ^
Group 2	8.5 ± 0.23^∗^	51.5 ± 2.39^∗^	5.2 ± 0.38^∗^	108.8 ± 7.65^∗^
Group 3	1.4 ± 0.15^ǂ^	121.8 ± 5.06^ǂ^	6.6 ± 0.31^∗^^ǂ^	90.7 ± 2.92
Group 4	1.4 ± 0.05^ǂ^	62.6 ± 1.98^∗^	7.2 ± 0.29^ǂ^	103.2 ± 3.65^∗^
Group 5	1.4 ± 0.04^ǂ^	63.1 ± 2.93^∗^	7.2 ± 0.19^ǂ^	100.0 ± 2.92^∗^
Group 6	1.9 ± 0.04^ǂ^	78.0 ± 3.17^∗^^ǂ^	7.1 ± 0.17^ǂ^	100.7 ± 6.96^∗^

Group 1: normal control; group 2: hypertensive control (L-NAME 50 mg/kg); group 3: L-NAME + lisinopril (10 mg/kg); group 4: L-NAME + Roselle-Olive (500 mg/kg); group 5: L-NAME + Roselle-Olive (250 mg/kg); group 6: L-NAME + Roselle-Olive (125 mg/kg). Values are expressed as mean ± SE (*n* = 7). ^∗^*P* < 0.05, statistically significant from the normal control group at the corresponding time. ^ǂ^*P* < 0.05, statistically significant from the hypertensive control group at the corresponding time.

**Table 5:** Real-time PCR quantitation of eNOS mRNA expression in the kidney and heart of the different experimental groups.

Groups	Fold change (RQ) in the kidney	Fold change (RQ) in the heart
Group 1	1^ǂ^	1^ǂ^
Group 2	0.14 ± 0.09^∗^	0.16 ± 0.08^∗^
Group 3	3.05 ± 1.12^∗^^ǂ^	6.67 ± 3.33^∗^^ǂ^
Group 4	3.97 ± 1.80^∗^^ǂ^	12.9 ± 4.60^∗^^ǂ^
Group 5	0.73 ± 0.11^ǂ^	10.11 ± 3.15^∗^^ǂ^
Group 6	0.16 ± 0.06^∗^	3.43 ± 2.83^∗^^ǂ^

Group 1: normal control; group 2: hypertensive control (L-NAME 50 mg/kg); group 3: L-NAME + lisinopril (10 mg/kg); group 4: L-NAME + Roselle-Olive (500 mg/kg); group 5: L-NAME + Roselle-Olive (250 mg/kg); group 6: L-NAME + Roselle-Olive (125 mg/kg). Values represent fold increases in mRNA level over the control group. GAPDH was used as an invariant internal control for calculating mRNA-fold changes. Values are expressed as mean ± SE. ^∗^*P* < 0.05, statistically significant from the normal control group at the corresponding time. ^ǂ^*P* < 0.05, statistically significant from the hypertensive control group at the corresponding time.

**Table 6:** Real-time PCR quantitation of iNOS mRNA expression in the kidney and heart of the different experimental groups.

Groups	Fold change (RQ) in the kidney	Fold change (RQ) in the heart
Group 1	1	1
Group 2	1.03 ± 0.25	0.96 ± 0.18
Group 3	0.95 ± 0.12	0.89 ± 0.37
Group 4	1.37 ± 0.41	1.45 ± 0.60
Group 5	1.33 ± 0.23	1.35 ± 0.35
Group 6	1.16 ± 0. 29	0.93 ± 0.43

Group 1: normal control; group 2: hypertensive control (L-NAME 50 mg/kg); group 3: L-NAME + lisinopril (10 mg/kg); group 4: L-NAME + Roselle-Olive (500 mg/kg); group 5: L-NAME + Roselle-Olive (250 mg/kg); group 6: L-NAME + Roselle-Olive (125 mg/kg). Values represent fold increases in mRNA level over the control group. GAPDH was used as an invariant internal control for calculating mRNA-fold changes. Values are expressed as mean ± SE. No significant differences between the examined groups.

**Table 7:** Tail length, tail intensity, and tail moment measured with Comet assay in the kidney of rats treated with L-NAME and protective influence of Roselle-Olive combination.

Groups	Comet %	Head diameter (*μ*m)	Tail length (*μ*m)	% DNA in tail	Tail moment
Group 1	9	38.7 ± 3.5^ǂ^	4.9 ± 1.2^ǂ^	26.9 ± 4.2^ǂ^	1.64 ± 0.35^ǂ^
Group 2	26.2	22.9 ± 2.9^∗^	6.87 ± 2.3^∗^	11.39 ± 1.7^∗^	2.87 ± 0.67^∗^
Group 3	19.4	32.1 ± 3.4^∗^^ǂ^	5.62 ± 1.9^∗^^ǂ^	13.36 ± 1.3 ^ǂ^	1.82 ± 0.72^ǂ^
Group 4	10.6	29.1 ± 2.9^ǂ^	4.2 ± 1.9^ǂ^	16.3 ± 2.6^∗^^ǂ^	1.45 ± 0.38^ǂ^
Group 5	11.6	31.8 ± 2.8^∗^^ǂ^	4.41 ± 1.6^ǂ^	14.4 ± 1.9^∗^	1.82 ± 0.82^ǂ^
Group 6	23.8	30.01 ± 2.5^∗^^ǂ^	4.2 ± 1.3^ǂ^	12.7 ± 1.2^∗^	1.6 ± 0.46^ǂ^

Group 1: normal control; group 2: hypertensive control (L-NAME 50 mg/kg); group 3: L-NAME + lisinopril (10 mg/kg); group 4: L-NAME + Roselle-Olive (500 mg/kg); group 5: L-NAME + Roselle-Olive (250 mg/kg); group 6: L-NAME + Roselle-Olive (125 mg/kg). Values are expressed as the mean ± SE. *n* = number of rats. ^∗^*P* < 0.05, statistically significant from the normal control group at the corresponding time. ^ǂ^*P* < 0.05, statistically significant from the hypertensive control group at the corresponding time.

**Table 8:** Tail length, tail intensity, and tail moment measured with Comet assay in the heart of rats treated with L-NAME and protective influence of Roselle-Olive combination.

Groups	Comet %	Head diameter (*μ*m)	Tail length (*μ*m)	% DNA in tail	Tail moment
Group 1	8	20.09 ± 3.1^ǂ^	5.2 ± 1.2^ǂ^	23.2 ± 2.8^ǂ^	1.31 ± 0.44^ǂ^
Group 2	24	14.3 ± 0.98^∗^	6.17 ± 2.2^∗^	30.6 ± 2.6^∗^	2.02 ± 0.89^∗^
Group 3	21.3	17.9 ± 1.6^∗^^ǂ^	5.9 ± 1.4^∗^	22.3 ± 3.3^ǂ^	1.82 ± 0.28^∗^^ǂ^
Group 4	19.7	17.2 ± 1.3^∗^^ǂ^	6.42 ± 1.9^∗^	23.3 ± 2.3^ǂ^	1.5 ± 0.56^ǂ^
Group 5	20.3	17.8 ± 1.2^∗^^ǂ^	5.81 ± 1.4^∗^^ǂ^	31.5 ± 2.8^∗^	1.89 ± 0.58^∗^
Group 6	22	14.9 ± 1.4^∗^	6.12 ± 1.5^∗^	31.5 ± 3.4^∗^	1.9 ± 0.72^∗^

Group 1: normal control; group 2: hypertensive control (L-NAME 50 mg/kg); group 3: L-NAME + lisinopril (10 mg/kg); group 4: L-NAME + Roselle-Olive (500 mg/kg); group 5: L-NAME + Roselle-Olive (250 mg/kg); group 6: L-NAME + Roselle-Olive (125 mg/kg). Values are expressed as the mean ± SE. *n* = number of rats. ^∗^*P* < 0.05, statistically significant from the normal control group at the corresponding time. ^ǂ^*P* < 0.05, statistically significant from the hypertensive control group at the corresponding time.

## Abbreviations

*HS*: *Hibiscus sabdariffa*
*OE*: *Olea europaea*
BP: Blood pressure
eNOS: Endothelial nitric oxide synthase
iNOS: Induced nitric oxide synthase
ROS: Reactive oxygen species
RNS: Reactive nitrogen species
LPO: Lipid peroxidation
MDA: Malondialdehyde
VSMCs: Vascular smooth muscle cells
ACE: Angiotensin-converting enzyme
L-NAME: N(G)-Nitro-L-arginine-methyl ester
RAS: Renin angiotensin system
Ang II: Angiotensin II.

## References

[1] Kahan T. (2014). Focus on blood pressure as a major risk factor. *The Lancet*.

[2] Staessen J. A. (2014). Hypertension: age-specificity of blood-pressure-associated complications. *Nature Reviews Cardiology*.

[3] World Health Organization (2003). 2003 World Health Organization (WHO)/International Society of Hypertension (ISH) statement on management of hypertension. *Journal of Hypertension*.

[4] Jing P., Qian B., He Y. (2014). Screening milk-derived antihypertensive peptides using quantitative structure activity relationship (QSAR) modelling and *in vitro/in vivo* studies on their bioactivity. *International Dairy Journal*.

[5] Pruijm M. T., Maillard M. P., Burnier M. (2008). Patient adherence and the choice of antihypertensive drugs: focus on lercanidipine. *Vascular Health and Risk Management*.

[6] Obouayeba A. P., Djyh N. B., Diabate S. (2014). Phytochemical and antioxidant activity of Roselle (*Hibiscus sabdariffa* L.) petal extracts. *Research Journal of Pharmaceutical, Biological and Chemical Sciences*.

[7] Anwar M. A., Al Disi S. S., Eid A. H. (2016). Anti-hypertensive herbs and their mechanisms of action: part II. *Frontiers in Pharmacology*.

[8] Da-Costa-Rocha I., Bonnlaender B., Sievers H., Pischel I., Heinrich M. (2014). *Hibiscus sabdariffa* L. – a phytochemical and pharmacological review. *Food Chemistry*.

[9] Dhar P., Kar C. S., Ojha D., Pandey S. K., Mitra J. (2015). Chemistry, phytotechnology, pharmacology and nutraceutical functions of kenaf (*Hibiscus cannabinus* L.) and roselle (*Hibiscus sabdariffa* L.) seed oil: an overview. *Industrial Crops and Products*.

[10] Ajiboye T. O., Salawu N. A., Yakubu M. T., Oladiji A. T., Akanji M. A., Okogun J. I. (2011). Antioxidant and drug detoxification potentials of *Hibiscus sabdariffa* anthocyanin extract. *Drug and Chemical Toxicology*.

[11] Mossalam H. H., Abd-El Aty O. A., Morgan E. N., Youssaf S. M., Mackawy A. M. (2011). Biochemical and ultra-structure studies of the antioxidant effect of aqueous extract of *Hibiscus sabdariffa* on the nephrotoxicity induced by organophosphorous pesticide (malathion) on the adult albino rats. *Journal of American Science*.

[12] Mohd-Esa N., Hern F. S., Ismail A., Yee C. L. (2010). Antioxidant activity in different parts of roselle (*Hibiscus sabdariffa* L.) extracts and potential exploitation of the seeds. *Food Chemistry*.

[13] Jonadet M., Bastide J., Bastide P., Boyer B., Carnat A. P., Lamaison J. L. (1990). *In vitro* enzyme inhibitory and *in vivo* cardioprotective activities of hibiscus (*Hibiscus sabdariffa* L.). *Journal de Pharmacie de Belgique*.

[14] Falade O. S., Otemuyiwa I. O., Oladipo A., Oyedapo O. O., Akinpelu B. A., Adewusi S. R. (2005). The chemical composition and membrane stability activity of some herbs used in local therapy for anemia. *Journal of Ethnopharmacology*.

[15] Peng C. H., Chyau C. C., Chan K. C., Chan T. H., Wang C. J., Huang C. N. (2011). *Hibiscus sabdariffa* polyphenolic extract inhibits hyperglycemia, hyperlipidemia, and glycation-oxidative stress while improving insulin resistance. *Journal of Agricultural and Food Chemistry*.

[16] Laikangbam R., Damayanti Devi M. D. (2012). Inhibition of calcium oxalate crystal deposition on kidneys of urolithiatic rats by *Hibiscus sabdariffa* L. extract. *Urological Research*.

[17] Gunstone F. (2002). *Vegetable Oils in Food Technology: Composition, Properties and Uses*.

[18] Kumral A., Giriş M., Soluk-Tekkeşin M. (2015). Effect of olive leaf extract treatment on doxorubicin-induced cardiac, hepatic and renal toxicity in rats. *Pathophysiology*.

[19] Perrinjaquet-Moccetti T., Busjahn A., Schmidlin C., Schmidt A., Bradl B., Aydogan C. (2008). Food supplementation with an olive (*Olea europaea* L.) leaf extract reduces blood pressure in borderline hypertensive monozygotic twins. *Phytotherapy Research*.

[20] Omar S. H. (2010). Cardioprotective and neuroprotective roles of oleuropein in olive. *Saudi Pharmaceutical Journal*.

[21] Carvajal-Zarrabal O., Barradas-Dermitz D. M., Orta-Flores Z. (2012). *Hibiscus sabdariffa* L., roselle calyx, from ethnobotany to pharmacology. *Journal of Experimental Pharmacology*.

[22] Susalit E., Agus N., Effendi I. (2011). Olive (*Olea europaea*) leaf extract effective in patients with stage-1 hypertension: comparison with Captopril. *Phytomedicine*.

[23] Majithiya J. B., Parmar A. N., Trivedi C. J., Balaraman R. (2005). Effect of pioglitazone on L-NAME induced hypertension in diabetic rats. *Vascular Pharmacology*.

[24] Meaney E., Alva F., Moguel R., Meaney A., Alva J., Webel R. (2000). Formula and nomogram for the sphygmomanometric calculation of the mean arterial pressure. *Heart*.

[25] Kushikata T., Hirota K., Yoshida H. (2003). Orexinergic neurons and barbiturate anesthesia. *Neuroscience*.

[26] Reitman S., Frankel S. (1957). A colorimetric method for the determination of serum glutamic oxalacetic and glutamic pyruvic transaminases. *American Journal of Clinical Pathology*.

[27] Szasz G. (1969). A kinetic photometric method for serum gamma-glutamyl transpeptidase. *Clinical Chemistry*.

[28] Wills M. R., Savory J. O. (1981). Biochemistry of renal failure. *Annals of Clinical & Laboratory Science*.

[29] Kroll M. H., Roach N. A., Poe B., Elin R. J. (1987). Mechanism of interference with the Jaffé reaction for creatinine. *Clinical Chemistry*.

[30] Placer Z. A., Cushman L. L., Johnson B. C. (1966). Estimation of product of lipid peroxidation (malonyl dialdehyde) in biochemical systems. *Analytical Biochemistry*.

[31] Miranda K. M., Espey M. G., Wink D. A. (2001). A rapid, simple spectrophotometric method for simultaneous detection of nitrate and nitrite. *Nitric Oxide*.

[32] Beutler E., Duron O., Kelly B. M. (1963). Improved method for the determination of blood glutathione. *The Journal of Laboratory and Clinical Medicine*.

[33] Friedewald W. T., Levy R. I., Fredrickson D. S. (1972). Estimation of the concentration of low-density lipoprotein cholesterol in plasma, without use of the preparative ultracentrifuge. *Clinical Chemistry*.

[34] Bancroft J. D., Gamble M. (2008). *Theory and Practice of Histological Techniques*.

[35] Duarte C. G., Zhang J., Ellis S. (1997). The SHR as a small animal model for radiocontrast renal failure. Relation of nephrotoxicity to animal’s age, gender, strain, and dose of radiocontrast. *Renal Failure*.

[36] Kanda T., Araki M., Nakano M. (1995). Chronic effect of losartan in a murine model of dilated cardiomyopathy: comparison with captopril. *Journal of Pharmacology and Experimental Therapeutics*.

[37] Ogaly H. A., Khalaf A. A., Ibrahim M. A., Galal M. K., Abd-Elsalam R. M. (2015). Influence of green tea extract on oxidative damage and apoptosis induced by deltamethrin in rat brain. *Neurotoxicology and Teratology*.

[38] Livak K. J., Schmittgen T. D. (2001). Analysis of relative gene expression data using real-time quantitative PCR and the 2−ΔΔCT method. *Methods*.

[39] Guo J. S., Cho C. H., Wang W. P., Shen X. Z., Cheng C. L., Koo M. W. (2003). Expression and activities of three inducible enzymes in the healing of gastric ulcers in rats. *World Journal of Gastroenterology*.

[40] Mostafa R. E., Salama A. A. A., Abdel-Rahman R. F., Ogaly H. A. (2017). Hepato and neuro-protective influences of biopropolis on thioacetamide-induced acute hepatic encephalopathy in rats. *Canadian Journal of Physiology and Pharmacology*.

[41] Eltablawy N. A., Ogaly H. A. (2014). Responsiveness of p53 expression and genetic mutation to CCl4-induced DNA damage in rat’s liver. *International Journal of Genomics and Proteomics*.

[42] Ramanathan V., Thekkumalai M. (2014). Role of chrysin on hepatic and renal activities of Nω-nitro-l-arginine-methylester induced hypertensive rats. *International Journal of Nutrition, Pharmacology, Neurological Diseases*.

[43] Pfeiffer S., Leopold E., Schmidt K., Brunner F., Mayer B. (1996). Inhibition of nitric oxide synthesis by NG-nitro-L-arginine methyl ester (L-NAME): requirement for bioactivation to the free acid, NG-nitro-L-arginine. *British Journal of Pharmacology*.

[44] Hopkins A. L., Lamm M. G., Funk J. L., Ritenbaugh C. (2013). *Hibiscus sabdariffa* L. in the treatment of hypertension and hyperlipidemia: a comprehensive review of animal and human studies. *Fitoterapia*.

[45] Jaarin K., Foong W. D., Yeoh M. H. (2015). Mechanisms of the antihypertensive effects of Nigella sativa oil in L-NAME-induced hypertensive rats. *Clinics*.

[46] Akinyemi A. J., Thome G. R., Morsch V. M. (2015). Effect of dietary supplementation of ginger and turmeric rhizomes on angiotensin-1 converting enzyme (ACE) and arginase activities in L-NAME induced hypertensive rats. *Journal of Functional Foods*.

[47] Cardoso A. M., Abdalla F. H., Bagatini M. D. (2014). Swimming training prevents alterations in acetylcholinesterase and butyrylcholinesterase activities in hypertensive rats. *American Journal of Hypertension*.

[48] Chillon J. M., Ghoneim S., Baumbach G. L. (1997). Effects of chronic nitric oxide synthase inhibition on cerebral arterioles in rats. *Hypertension*.

[49] Baumbach G. L., Heistad D. D., Siems J. E. (1989). Effect of sympathetic nerves on composition and distensibility of cerebral arterioles in rats. *The Journal of Physiology*.

[50] Ojeda D., Jiménez-Ferrer E., Zamilpa A., Herrera-Arellano A., Tortoriello J., Alvarez L. (2010). Inhibition of angiotensin convertin enzyme (ACE) activity by the anthocyanins delphinidin-and cyanidin-3-O-sambubiosides from *Hibiscus sabdariffa*. *Journal of Ethnopharmacology*.

[51] Hansen K., Adsersen A., Christensen S. B., Jensen S. R., Nyman U., Smitt U. W. (1996). Isolation of an angiotensin converting enzyme (ACE) inhibitor from *Olea europaea* and *Olea lancea*. *Phytomedicine*.

[52] Aliyu B., Oyeniyi Y. J., Mojiminiyi F. B., Isezuo S. A., Alada A. R. (2014). The aqueous calyx extract of *Hibiscus sabdariffa* lowers blood pressure and heart rate via sympathetic nervous system dependent mechanisms. *Nigerian Journal of Physiological Sciences*.

[53] Micucci M., Malaguti M., Gallina Toschi T. (2015). Cardiac and Vascular Synergic Protective Effect of *Olea europaea* L. Leaves and *Hibiscus sabdariffa* L. Flower Extracts. *Oxidative Medicine and Cellular Longevity*.

[54] Chaswal M., Das S., Prasad J., Katyal A., Mishra A. K., Fahim M. (2012). Effect of losartan, an angiotensin II type 1 receptor antagonist on cardiac autonomic functions of rats during acute and chronic inhibition of nitric oxide synthesis. *Physiological Research*.

[55] Silva V. J., Ferreira Neto E., Salgado H. C., Fazan Júnior R. (2002). Chronic converting enzyme inhibition normalizes QT interval in aging rats. *Brazilian Journal of Medical and Biological Research*.

[56] Cohuet G., Struijker-Boudier H. (2006). Mechanisms of target organ damage caused by hypertension: therapeutic potential. *Pharmacology and Therapeutics*.

[57] Vardi N., Ozturk F., Fadillioglu E., Otlu A., Yagmurca M. (2003). Histological changes in the rat thoracic aorta after chronic nitric oxide synthase inhibition. *Turkish Journal of Medical Sciences*.

[58] Rossoni G., Manfredi B., Gennaro Colonna V., Berti M., Guazzi M., Berti F. (2007). Sildenafil reduces L-NAME-induced severe hypertension and worsening of myocardial ischaemia-reperfusion damage in the rat. *British Journal of Pharmacology*.

[59] Adaramoye O. A., Nwosu I. O., Farombi E. O. (2012). Sub-acute effect of NG-nitro-l-arginine methyl-ester (L-NAME) on biochemical indices in rats: protective effects of Kolaviron and extract of *Curcuma longa* L. *Pharmacognosy Research*.

[60] Talas Z. S., Gogebakan A., Orun I. (2013). Effects of propolis on blood biochemical and hematological parameters in nitric oxide synthase inhibited rats by Nω-nitro-L-arginine methyl ester. *Pakistan Journal of Pharmaceutical Sciences*.

[61] Barón V., Hernández J., Noyola M., Escalante B., Muriel P. (2000). Nitric oxide and inducible nitric oxide synthase expression are downregulated in acute cholestasis in the rat accompanied by liver ischemia. *Comparative Biochemistry and Physiology Part C: Pharmacology, Toxicology and Endocrinology*.

[62] Cottart C. H., Nivet-Antoine V., Do L. (2003). Hepatic cytoprotection by nitric oxide and the cGMP pathway after ischaemia–reperfusion in the rat. *Nitric Oxide*.

[63] Lee C. H., Kuo C. Y., Wang C. J. (2012). A polyphenol extract of *Hibiscus sabdariffa* L. ameliorates acetaminophen-induced hepatic steatosis by attenuating the mitochondrial dysfunction in vivo and in vitro. *Bioscience, Biotechnology, and Biochemistry*.

[64] Giannini E. G., Testa R., Savarino V. (2005). Liver enzyme alteration: a guide for clinicians. *Canadian Medical Association Journal*.

[65] Mount P. F., Power D. A. (2006). Nitric oxide in the kidney: functions and regulation of synthesis. *Acta Physiologica*.

[66] Ali B. H., Wabel N. A., Blunden G. (2005). Phytochemical, pharmacological and toxicological aspects of *Hibiscus sabdariffa* L.: a review. *Phytotherapy Research*.

[67] Alarcón-Alonso J., Zamilpa A., Aguilar F. A., Herrera-Ruiz M., Tortoriello J., Jimenez-Ferrer E. (2012). Pharmacological characterization of the diuretic effect of *Hibiscus sabdariffa* Linn (Malvaceae) extract. *Journal of Ethnopharmacology*.

[68] Nyadjeu P., Nguelefack-Mbuyo E. P., Atsamo A. D., Nguelefack T. B., Dongmo A. B., Kamanyi A. (2013). Acute and chronic antihypertensive effects of *Cinnamomum zeylanicum* stem bark methanol extract in L-NAME-induced hypertensive rats. *BMC Complementary and Alternative Medicine*.

[69] Chen C. C., Hsu J. D., Wang S. F. (2003). *Hibiscus sabdariffa* extract inhibits the development of atherosclerosis in cholesterol-fed rabbits. *Journal of Agricultural and Food Chemistry*.

[70] Onyenekwe P. C., Ajani E. O., Ameh D. A., Gamaniel K. S. (1999). Antihypertensive effect of roselle (*Hibiscus sabdariffa*) calyx infusion in spontaneously hypertensive rats and a comparison of its toxicity with that in Wistar rats. *Cell Biochemistry and Function*.

[71] Atlas S. A. (2007). The renin-angiotensin aldosterone system: pathophysiological role and pharmacologic inhibition. *Journal of Managed Care Pharmacy*.

[72] Ng K. K., Vane J. R. (1967). Conversion of angiotensin I to angiotensin II. *Nature*.

[73] Singh P., Deng A., Weir M. R., Blantz R. C. (2008). The balance of angiotensin II and nitric oxide in kidney diseases. *Current Opinion in Nephrology and Hypertension*.

[74] Giani J. F., Janjulia T., Kamat N. (2014). Renal angiotensin-converting enzyme is essential for the hypertension induced by nitric oxide synthesis inhibition. *Journal of the American Society of Nephrology*.

[75] Mojiminiyi F. B., Dikko M., Muhammad B. Y. (2007). Antihypertensive effect of an aqueous extract of the calyx of *Hibiscus sabdariffa*. *Fitoterapia*.

[76] Nekooeian A. A., Dehghani G. A., Mostafavi H., Khalili A. (2011). The effect of hydroalcoholic extract of olive leaves on blood pressure in rat model of two-kidney, one-clip goldblatt hypertension. *International Cardivascular Research Journal*.

[77] Khayyal M. T., El-Ghazaly M. A., Abdallah D. M., Nassar N. N., Okpanyi S. N., Kreuter M. H. (2002). Blood Pressure Lowering Effect of an Olive Leaf Extract (*Olea europaea*) in L-NAME Induced Hypertension in Rats. *Arzneimittelforschung*.

[78] Zhou M. S., Schulman I. H., Raij L. (2004). Nitric oxide, angiotensin II, and hypertension. *Seminars in Nephrology*.

[79] Montezano A. C., Dulak-Lis M., Tsiropoulou S., Harvey A., Briones A. M., Touyz R. M. (2015). Oxidative stress and human hypertension: vascular mechanisms, biomarkers, and novel therapies. *Canadian Journal of Cardiology*.

[80] Forte M., Conti V., Damato A. (2016). Targeting nitric oxide with natural derived compounds as a therapeutic strategy in vascular diseases. *Oxidative Medicine and Cellular Longevity*.

[81] Selemidis S., Dusting G. J., Peshavariya H., Kemp-Harper B. K., Drummond G. R. (2007). Nitric oxide suppresses NADPH oxidase-dependent superoxide production by S-nitrosylation in human endothelial cells. *Cardiovascular Research*.

[82] Sung J. H., Jo Y. S., Kim S. J. (2013). Effect of lutein on L-NAME-induced hypertensive rats. *The Korean Journal of Physiology and Pharmacology*.

[83] Öktem F., Kirbas A., Armagan A. (2011). Lisinopril attenuates renal oxidative injury in L-NAME-induced hypertensive rats. *Molecular and Cellular Biochemistry*.

[84] Ogaly H. A., Eltablawy N. A., El-Behairy A. M., El-Hindi H., Abd-Elsalam R. M. (2015). Hepatocyte growth factor mediates the antifibrogenic action of *Ocimum bacilicum* essential oil against CCl4-induced liver fibrosis in rats. *Molecules*.

[85] Čabarkapa A., Živković L., Žukovec D. (2014). Protective effect of dry olive leaf extract in adrenaline induced DNA damage evaluated using *in vitro* comet assay with human peripheral leukocytes. *Toxicology In Vitro*.

[86] Fabiani R., Rosignoli P., De Bartolomeo A. (2008). Oxidative DNA damage is prevented by extracts of olive oil, hydroxytyrosol, and other olive phenolic compounds in human blood mononuclear cells and HL60 cells. *The Journal of Nutrition*.

[87] Ferroni P., Basili S., Paoletti V., Davi G. (2006). Endothelial dysfunction and oxidative stress in arterial hypertension. *Nutrition, Metabolism and Cardiovascular Diseases*.

[88] Nakmareong S., Kukongviriyapan U., Pakdeechote P. (2012). Tetrahydrocurcumin alleviates hypertension, aortic stiffening and oxidative stress in rats with nitric oxide deficiency. *Hypertension Research*.

[89] Zhao Y., Vanhoutte P. M., Leung S. W. (2013). Endothelial nitric oxide synthase-independent release of nitric oxide in the aorta of the spontaneously hypertensive rat. *Journal of Pharmacology and Experimental Therapeutics*.

[90] Giani J. F., Janjulia T., Taylor B. (2014). Renal generation of angiotensin II and the pathogenesis of hypertension. *Current Hypertension Reports*.

[91] Pushpakumar S. B., Kundu S., Metreveli N., Sen U. (2013). Folic acid mitigates angiotensin-II-induced blood pressure and renal remodeling. *PLoS One*.

[92] Monhart V. (2013). Hypertension and chronic kidney diseases. *Cor et Vasa*.

[93] Biwer L. A., D'souza K. M., Abidali A. (2016). Time course of cardiac inflammation during nitric oxide synthase inhibition in SHR: impact of prior transient ACE inhibition. *Hypertension Research*.

[94] Yu Q., Horak K., Larson D. F. (2006). Role of T lymphocytes in hypertension-induced cardiac extracellular matrix remodeling. *Hypertension*.

[95] Hale T. M., Robertson S. J., Burns K. D., deBlois D. (2012). Short-term ACE inhibition confers long-term protection against target organ damage. *Hypertension Research*.

[96] Chou T. C., Yen M. H., Li C. Y., Ding Y. A. (1998). Alterations of nitric oxide synthase expression with aging and hypertension in rats. *Hypertension*.

[97] Francis S. H., Busch J. L., Corbin J. D. (2010). cGMP-dependent protein kinases and cGMP phosphodiesterases in nitric oxide and cGMP action. *Pharmacological Reviews*.

[98] Fadel P. J. (2017). Nitric oxide and cardiovascular regulation. *Hypertension*.

[99] Melikian N., Seddon M. D., Casadei B., Chowienczyk P. J., Shah A. M. (2009). Neuronal nitric oxide synthase and human vascular regulation. *Trends in Cardiovascular Medicine*.

[100] Rodriguez-Rodriguez R., Herrera M. D., De Sotomayor M. A., Ruiz-Gutierrez V. (2007). Pomace olive oil improves endothelial function in spontaneously hypertensive rats by increasing endothelial nitric oxide synthase expression. *American Journal of Hypertension*.

[101] Ajay M., Chai H. J., Mustafa A. M., Gilani A. H., Mustafa M. R. (2007). Mechanisms of the anti-hypertensive effect of *Hibiscus sabdariffa* L. calyces. *Journal of Ethnopharmacology*.

[102] Lim Y. C., Budin S. B., Othman F., Latip J., Zainalabidin S. (2017). Roselle polyphenols exert potent negative inotropic effects via modulation of intracellular calcium regulatory channels in isolated rat heart. *Cardiovascular Toxicology*.

[103] Pechánová O., Dobešová Z., Cejka J., Kuneš J., Zicha J. (2004). Vasoactive systems in L-NAME hypertension: the role of inducible nitric oxide synthase. *Journal of Hypertension*.

[104] Santhanam L., Lim H. K., Lim H. K. (2007). Inducible NO synthase dependent S-nitrosylation and activation of arginase1 contribute to age-related endothelial dysfunction. *Circulation Research*.

[105] Oliveira-Paula G. H., Lacchini R., Tanus-Santos J. E. (2014). Inducible nitric oxide synthase as a possible target in hypertension. *Current Drug Targets*.

[106] Meira L. B., Bugni J. M., Green S. L. (2008). DNA damage induced by chronic inflammation contributes to colon carcinogenesis in mice. *The Journal of Clinical Investigation*.

[107] Ghosh I., Poddar S., Mukherjee A. (2015). Evaluation of the protective effect of *Hibiscus sabdariffa* L. calyx (Malvaceae) extract on arsenic induced genotoxicity in mice and analysis of its antioxidant properties. *Biology and Medicine*.

[108] Abdul Hamid Z., Lin Lin W. H., Abdalla B. J. (2014). The role of *Hibiscus sabdariffa* L. (Roselle) in maintenance of ex vivo murine bone marrow-derived hematopoietic stem cells. *The Scientific World Journal*.

[109] Machowetz A., Poulsen H. E., Gruendel S. (2007). Effect of olive oils on biomarkers of oxidative DNA stress in northern and southern Europeans. *The FASEB Journal*.

